# Bispecific T cell engagers for treatment-refractory autoimmune connective tissue diseases

**DOI:** 10.1038/s41591-026-04238-4

**Published:** 2026-02-19

**Authors:** Christina Düsing, Andrea-Hermina Györfi, Ayla Nadja Stütz, Laura-Marie Lahu, Franca Sophie Deicher, Yi-Nan Li, Peter-Martin Bruch, Alexandru-Emil Matei, Alexandru Micu, Tim Filla, Sarah Koziel, Alexander Sebastian Hölscher, Manuel Röhrich, Hanns-Martin Lorenz, Mareike Cramer, Gerald Antoch, Jörg Timm, Yousef Abusabha, Tobias Ruck, Bernhard Homey, Alexander Kreuter, Björn Buehring, Aleksandar Radujkovic, Claus Peter Heußel, Melanie Hagen, Ricardo Grieshaber-Bouyer, Georg Schett, Anna Brunn, Sascha Dietrich, Wolfgang Merkt, Jörg H. W. Distler

**Affiliations:** 1 Department of Rheumatology, University Hospital Düsseldorf, Medical Faculty of Heinrich-Heine University, Düsseldorf, Germany; 2 Hiller Research Center, University Hospital Düsseldorf, Medical Faculty of Heinrich-Heine University, Düsseldorf, Germany; 3 Fraunhofer Institute for Translational Medicine and Pharmacology ITMP, and Fraunhofer Cluster of Excellence for Immune Mediated Diseases CIMD, Frankfurt am Main, Germany; 4 Spatial & Functional Screening Core Facility, Medical Faculty, Heinrich Heine University, Düsseldorf, Germany; 5 Center for Integrated Oncology Aachen Bonn Cologne Düsseldorf (CIO ABCD), Düsseldorf, Germany; 6 Molecular Medicine Partnership Unit, Heidelberg, Germany; 7 Department of Hematology, Oncology and Clinical Immunology, Heinrich-Heine University, Düsseldorf, Germany; 8 Department of Nuclear Medicine, Universitätsmedizin Mainz, Mainz, Germany; 9 Division of Rheumatology, Department of Hematology, Oncology and Rheumatology, Internal Medicine V, University Hospital Heidelberg, Heidelberg, Germany; 10 Department of Cardiology, Pneumology, and Angiology, University Hospital Düsseldorf, Medical Faculty of Heinrich-Heine University, Düsseldorf, Germany; 11 Department of Diagnostic and Interventional Radiology, Medical Faculty, Heinrich-Heine University Düsseldorf, Düsseldorf, Germany; 12 Institute of Virology, University Hospital Düsseldorf, Medical Faculty of Heinrich-Heine University, Düsseldorf, Germany; 13 Department of Neurosurgery, Medical Faculty, Heinrich-Heine University Düsseldorf, Düsseldorf, Germany; 14 BG University Hospital Bergmannsheil, Heimer Institute for Muscle Research, Bochum, Germany; 15 Department of Neurology, Ruhr University Bochum, BG University Hospital Bergmannsheil, Bochum, Germany; 16 Department of Dermatology, University Hospital Düsseldorf, Medical Faculty of Heinrich-Heine University, Düsseldorf, Germany; 17 Department of Dermatology, Venereology and Allergology, HELIOS St. Elisabeth Clinic Oberhausen, University Witten-Herdecke, Oberhausen, Germany; 18 Department of Dermatology, Venereology und Allergology, Helios St. Johannes Clinic Duisburg, Duisburg, Germany; 19 Department of Internal Medicine and Rheumatology, Cellitinnen Hospital St. Josef, Wuppertal, Germany; 20 Department of Diagnostic and Interventional Radiology, University Hospital Heidelberg, Heidelberg, Germany; 21 Translational Lung Research Center Heidelberg (TLRC), German Center for Lung Research (DZL), Heidelberg, Germany; 22 Department of Diagnostic and Interventional Radiology with Nuclear Medicine, Thoraxklinik at the University Hospital of Heidelberg, Heidelberg, Germany; 23 Department of Internal Medicine 3, Rheumatology and Clinical Immunology, Friedrich-Alexander University (FAU) Erlangen-Nürnberg and Universitätsklinikum Erlangen, Erlangen, Germany; 24 Deutsches Zentrum Immuntherapie (DZI), Friedrich-Alexander University (FAU) Erlangen-Nürnberg and Universitätsklinikum Erlangen, Erlangen, Germany; 25 Institute of Neuropathology, Medical Faculty, Heinrich-Heine University and University Hospital Düsseldorf, Düsseldorf, Germany

**Keywords:** Autoimmune diseases, Immunotherapy

## Abstract

Autoimmune-mediated connective tissue diseases such as antisynthetase syndrome (ASyS) and systemic sclerosis (SSc) have a high unmet medical need. Here we report on treatment under compassionate use with the CD19×CD3 T cell engager (TCE) blinatumomab and the BCMA×CD3 TCE teclistamab in five patients with treatment-refractory ASyS and in five patients with treatment-refractory SSc, respectively. Induction therapy with blinatumomab or teclistamab reduced target cells in affected muscle and skin, respectively, and decreased autoantibody titers. Blinatumomab induced rapid clinical, serological and histological improvement of myositis and stabilization of interstitial lung disease (ILD) in patients with ASyS. Teclistamab improved skin fibrosis, stabilized ILD and resolved tendon friction rubs in patients with SSc. Inhibition of B cell redifferentiation by maintenance therapy with rituximab (RTX) enabled prolonged disease control, even for patients previously unresponsive to RTX. Treatment was associated with adverse events including cytokine release syndrome (CRS) up to grade 3, in two patients with ASyS and in all patients with SSc. No immune effector cell-associated neurotoxicity syndrome (ICANS) occurred. Respiratory infections treated with antibiotics occurred in six patients. Blinatumomab and teclistamab may offer potential as rescue therapies for patients with treatment-refractory ASyS and SSc.

## Abstract (web summary)

In a case series of five patients with treatment-refractory antisynthetase syndrome and five patients with treatment-refractory systemic sclerosis, bispecific T cell engagers blinatumomab and teclistamab improved disease activity and were well tolerated.

## Main

Connective tissue diseases are characterized by activation of B cells and the presence of specific autoantibodies. Autoantibodies are not only markers of individual diseases but can also activate disease-associated molecular pathways^1–5^. B cells also drive the pathogenesis of autoimmune diseases by release of inflammatory cytokines, by antigen presentation and by promoting macrophage activation^6^. ASyS and SSc are two autoimmune-mediated connective tissue diseases with particularly high medical need for novel therapies.

ASyS is a subtype of idiopathic inflammatory myopathy characterized by autoantibodies against aminoacyl-tRNA synthetases^7^. Anti-Jo1 autoantibodies are the most frequent ASyS-specific autoantibodies, present in approximately 60−70% of patients with ASyS. The B-cell-depleting anti-CD20 monoclonal antibody RTX is used as an off-label therapy in ASyS^8^. Recent reports on CD19 chimeric antigen receptor (CAR)-T cells and teclistamab for the treatment of ASyS have sparked interest in alternative B-cell-depleting approaches^9–14^. However, application of autologous, ex vivo-generated CD19-targeting CAR-T cell therapies requires specific infrastructure and has other disadvantages, such as treatment delay due to CAR-T cell production, potential toxicity of the preconditioning chemotherapy and excessive costs. Alternative approaches may facilitate broader application of deep depletion of CD19^+^ B cells for the treatment of ASyS.

SSc is characterized by autoimmunity, vasculopathy and fibrosis^15,16^. Anti-topoisomerase 1 and anti-fibrillarin autoantibodies characterize subgroups of patients with diffuse-cutaneous SSc (dcSSc) with severe organ involvement and poor prognosis^17,18^. B cell targeting by RTX demonstrated clinical efficacy on dermal and pulmonary fibrosis^19–21^. Furthermore, first reports indicate that CD19-targeting CAR-T cells may offer improved therapeutic potential in dcSSc^22–25^. However, anti-topoisomerase 1 autoantibodies remained detectable in patients with SSc treated with second-generation CD19-targeting CAR-T cells, suggesting that CD19-targeting strategies do not eliminate anti-topoisomerase autoantibody-producing B cells.

Currently available therapies that specifically deplete B cells are directed against CD19, CD20 or B cell maturation antigen (BCMA). CD19 is expressed across a broader range of B cell developmental stages than CD20, allowing depletion of additional B cell populations, including plasmablasts. However, long-lived plasma cells are not effectively targeted by CD19-targeted therapies. BCMA is highly expressed on plasma cells. BCMA-directed therapies may, thus, enable more effective targeting of autoantibody production than CD19-directed and CD20-directed therapies.

TCEs are an emerging class of therapeutics. These bispecific monoclonal antibody derivates bind to antigens on target cells as well as T cells, leading to T cell activation and killing of the target cell by T-cell-mediated cytotoxicity^26,27^. Blinatumomab binds CD19 and CD3 and is currently approved for the treatment of acute lymphoblastic leukemia. Teclistamab binds BCMA and CD3 and is approved for the treatment of multiple myeloma. First single cases and case series describing the use of blinatumomab^14,28^ and of teclistamab in autoimmune diseases have recently been published^29–31^.

In the present case series, we describe the efficacy and safety of blinatumomab and teclistamab as an induction therapy in five patients with ASyS and in five patients with SSc, respectively. All patients had severe, advanced, progressive disease and previously did not achieve disease control with at least three immunomodulatory or antifibrotic therapies, including RTX in all patients with ASyS and in three patients with SSc. We also report on the efficacy of an RTX-based regimen as subsequent maintenance therapy, aiming to prevent replenishment of tissue niches by pathogenic B cells after TCE-induced depletion.

## Results

**Extended Data Fig. 1:**
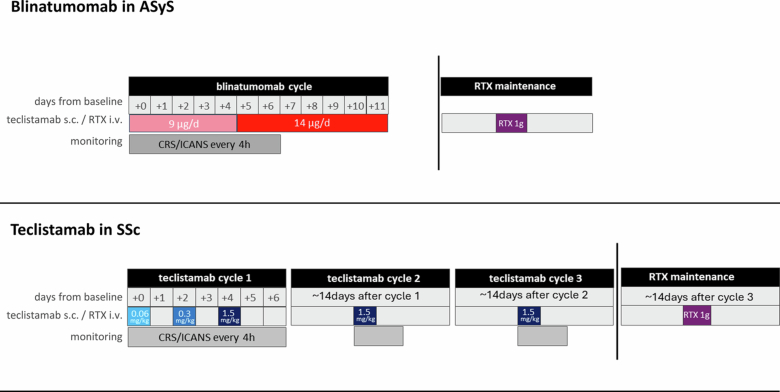
Schematic representation of blinatumomab and teclistamab treatment. Top: Schematic representation of induction therapy with blinatumomab and maintenance therapy with RTX treatment in patients with ASyS including dosing and monitoring. Bottom: Schematic representation of induction therapy with teclistamab and maintenance therapy with RTX treatment in patients with SSc including dosing and monitoring. *Abbreviations*: CRS, cytokine release syndrome; ICANS, immune effector cell-associated neurotoxicity syndrome; RTX, Rituximab.

Patient characteristics at baseline are described in the Methods section and in Table 1 (patients 1−5 with ASyS) and Table 2 (patients 6−10 with SSc). All patients were treatment refractory and progressed despite at least three previous different treatment attempts. All patients with ASyS (patients 1−5) were positive for anti-Jo1 autoantibodies, and four of five patients with SSc were positive for anti-topoisomerase 1 autoantibodies. Patients with ASyS received an induction therapy with blinatumomab and patients with SSc with teclistamab, followed by maintenance therapy with RTX. Schematic representation of the treatment schedules is shown in Extended Data Fig. 1.

**Table 1:** Demographics and clinical characteristics of patients with ASyS at baseline

Characteristics	Patient 1	Patient 2	Patient 3	Patient 4	Patient 5
General					
Age (years)	65	37	47	48	50
Sex	Female	Male	Male	Female	Female
Disease duration (years)	2	1.5	4	5	5
Autoantibodies					
Anti-Jo1	+	+	+	+	+
Others	-	Ro-52	-	Ro-52, PM-100, AMA-M2	Ro-52
Lung					
ILD	+	+	-	+	+
HRCT	NSIP	NSIP	-	NSIP/OP	NSIP/OP
FVC, % predicted	65	38	74	30	52
DLCO, % predicted	71	27	86	70	82
History of smoking (PY)	0	0	0	15	5
6MWD (m)	200	0	440	N/A	375
Oxygen (l min^−1^)	0	2−6	0	4	0
Heart					
pHI on CMR	-	+	-	+	-
LV-EF (%)	66	63	43	63	61
Musculoskeletal					
Myositis	+	+	+	+	+
Arthritis	-	+	-	-	+
Skin					
Mechanic’s hands	+	+	+	+	-
Previous immunomodulatory/antifibrotic therapies					
MTX	+	-	+	+	+
Mycophenolate	-	+	-	+	+
Azathioprin	+	-	+	-	+
Cyclophosphamide	-	+	-	+	-
RTX	+	+	+	+	+
IVIG	+	-	+	+	+
Tofacitinib	-	+	+	-	-
Others		Nintedanib, tacrolimus, certolizumab, tofacitinib	Hydroxy-chloroquine	Ciclosporin, hydroxy-chloroquine	Hydroxy-chloroquine
Relevant comorbidities					
	Breast cancer, PE, hepatitis B	Depression	PSC, UC, PE, *M. kansasii*	Amputation, ARDS, epilepsy	ARDS (invasive ventilation, ECMO)

ECMO, extracorporeal membrane oxygenation; HRCT, high-resolution computed tomography of the chest; N/A, not applicable; OP, organizing pneumonia; PE, pulmonary embolism; PSC, primary sclerosing cholangitis; PY, pack-years; UC, ulcerative colitis.

**Table 2:** Demographics and clinical characteristics of patients with SSc at baseline

Characteristics	Patient 6	Patient 7	Patient 8	Patient 9	Patient 10
General					
Age (years)	65	52	56	46	46
Sex	Male	Male	Male	Male	Male
Inflammatory subtype*	-	+	+	+	+
Disease duration (years)	18	2	4	3	1
Autoantibodies					
Anti-topoisomerase 1	+	+	+	+	-
Others	-	-	-	-	Anti-fibrillarin; ACPA
Skin					
dcSSc	+	+	+	+	+
mRSS	21	22	28	38	23
Lung: SSc-ILD					
CT pattern	cNSIP	CPFE	cNSIP	NSIP	NSIP
FVC, % predicted	52	80	52	68	54
DLCO-VA, % predicted	63	75	90	104	83
History of smoking (PY)	0	80**	0	40	10
Heart: SSc-pHI					
Pathologic CMR	+	+	+	+	+
LV-EF (%)	57	61	67	41	54
ECG abnormalities	+	+	+	+	-
Increased hsTnT	+	+	+	+	+
GI					
Upper GI***	+	+	+	+	+
Weight loss	14 kg / 10 years	15 kg / 1 year	10 kg / 4 years	10 kg / 2 years	18 kg / 1 year
Musculoskeletal					
Arthritis and myositis	-	-	-	+	+
TFRs	3	6	3	+	12
Vascular					
Ischemic ulcers	Pitting scars	+	+	+	-
SSc-PH	-	-	-	-	-
Previous immunomodulatory/antifibrotic therapies					
Mycophenolate	+	+	+	-	+
MTX	-	+	+	+	-
Nintedanib	-	-	+	-	+
Cyclophosphamide	+	-	-	+	+
Tocilizumab	-	-	-	-	-
RTX	-	-	+	+	+
Others	Aza-thioprine	Hydroxy-chloroquine	-	-	-

ACPA, anticitrullinated protein antibody; COPD, chronic obstructive lung disease; cNSIP, cellular non-specific interstitial pneumonia; CPFE, combined pulmonary fibrosis and emphysema; CT, computed tomography; ECG, electrocardiogram; GI, gastrointestinal; PY, pack-years; SSc-PH, SSc-associated pulmonary hypertension. * Inflammatory subtype was defined according to the randomized clinical trials with tocilizumab in SSc. ** This patient stopped smoking immediately prior to initiation of teclistamab. *** According to reported symptoms such as reflux and dysphagia and findings in CT imaging and endoscopy.

### Clinical outcome of patients with ASyS receiving induction therapy with blinatumomab and maintenance therapy with RTX

**Fig. 1:**
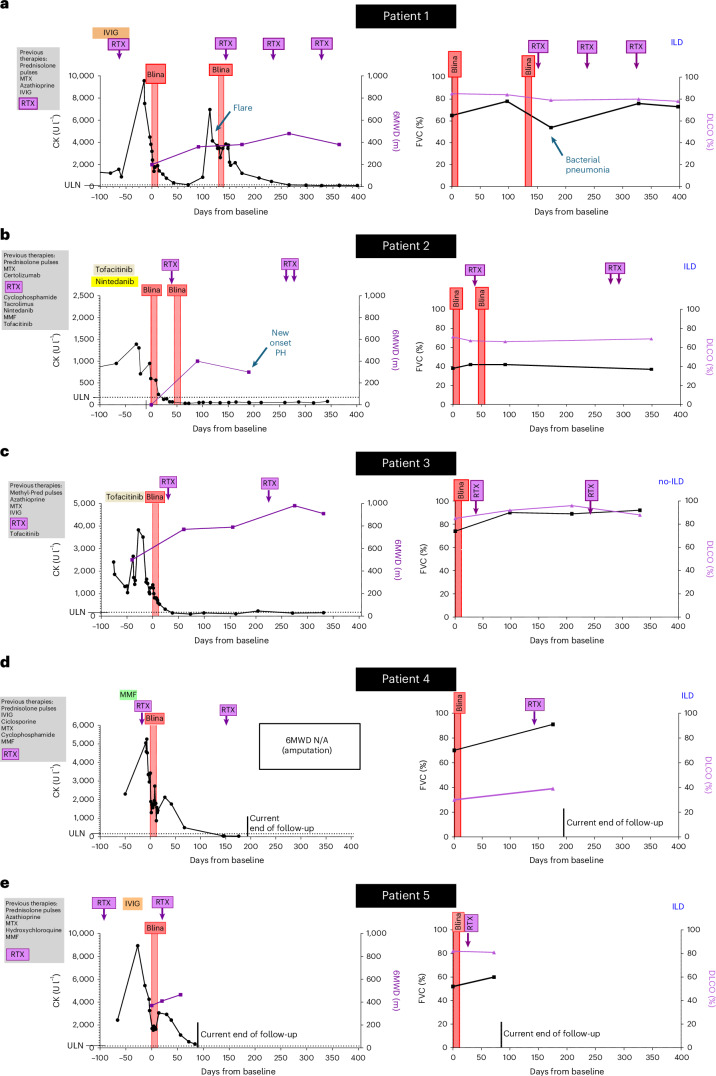
Clinical outcomes of individual patients with ASyS in response to induction therapy with blinatumomab and maintenance therapy with RTX. **a**−**e**, Treatment history and changes in CK levels and 6MWD in individual patients with ASyS (left) and changes of FVC (percent predicted) and DLCO_VA (DLCO, percent predicted) (right). Blina, blinatumomab; CK, creatine kinase (normal range: 0−171 U l^−1^); N/A, not applicable.

**Extended Data Fig. 2:**
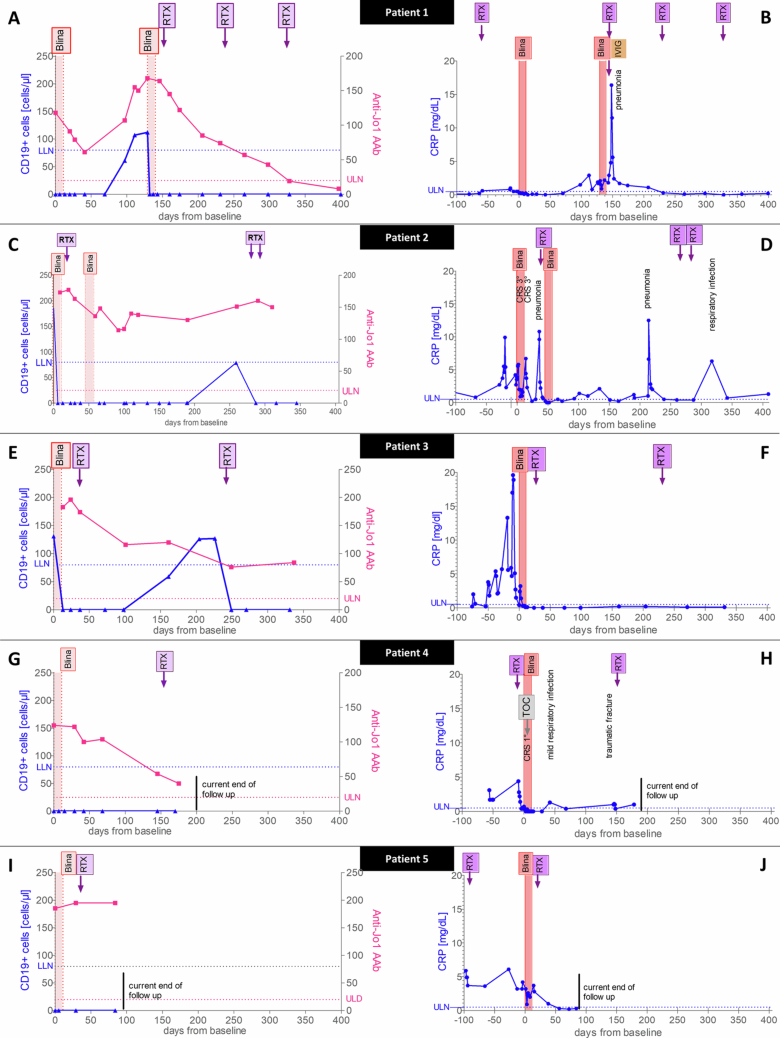
Changes of CD19+ cells and anti-Jo1 autoantibody levels or CRP after induction therapy with blinatumomab and maintenance therapy with RTX in patients with ASyS. **A**, **C**, **E**, **G**, **I**: Changes in CD19 + B cells [normal range: 73 – 562/µl] and anti-Jo1 autoantibodies after blinatumomab and RTX treatment in patients 1 – 5 with ASyS. **B**, **D**, **F**, **H**, **J** Changes in CRP levels [normal range: <0.5 mg/dl] and relevant AEs. *Abbreviations*: RTX, Rituximab; AAb, autoantibody; CRP, C-reactive protein; IVIG, intravenous immunoglobulins; AE, adverse events; CRS, cytokine release syndrome; LLN, lower limit of normal; ULN, upper limit of normal; TOC, tocilizumab.

Patient 1 (ASyS): Within 3 months of blinatumomab induction, patient 1 demonstrated sustained clinical and physiological improvement with declining creatine kinase (CK) levels (Fig. 1a) and an increase in Manual Muscle Testing 8 (MMT8) score (142 versus 118 at baseline) and in 6-minute walking distance (6MWD). The patient’s dyspnea improved from New York Heart Association (NYHA) III to NYHA I; forced vital capacity (FVC) improved slightly; diffusing lung capacity for carbon monoxide (DLCO) remained stable (Fig. 1a); and mechanic’s hands resolved. The total improvement score (TIS) reached 70% after 3 months. Afterwards, however, an increase in CD19^+^ B cells in blood was observed with increases in anti-Jo1 autoantibody titers and C-reactive protein (CRP) (Extended Data Fig. 2a,b). This was accompanied by recurrence of myositis, evidenced by rising CK levels, inflammatory changes in the quadriceps muscles on magnetic resonance imaging (MRI), worsening dyspnea and a decline in FVC. We retreated the patient with blinatumomab followed by RTX this time. She rapidly responded with normalization of CK levels and improvement of FVC (Fig. 1a). MRI confirmed regression of myositis 3 months after reinduction therapy. With repetitive RTX infusions, B cells remained fully suppressed, the titers of anti-Jo1 autoantibody continuously declined and the patient maintained glucocorticoid-free disease control throughout the remaining follow-up period, reaching a TIS of 80% 12 months after baseline (Extended Data Fig. 2a). MRI of both thighs after 9 months demonstrated persistent resolution of myositis (Supplementary Fig. 1a).

Patient 2 (ASyS): Within 1 month after blinatumomab, CK levels normalized (Fig. 1b), N-terminal pro-B-type natriuretic peptide (NT-proBNP) declined, 6MWD improved (398 m versus 0 m at baseline), dyspnea improved to NYHA II (versus NYHA III/IV at baseline), FVC and DLCO remained stable (Fig. 1b) and mechanic’s hands resolved. Given the advanced ILD, overall fragility and the relapse in patient 1, we applied another course of blinatumomab starting at day 39, aiming for a more durable response. After 3 months, TIS reached 60%. Four months after induction therapy with blinatumomab, MRI of the right upper arm demonstrated resolution of myositis (Supplementary Fig. 1b). High-resolution computed tomography (HRCT) demonstrated reduced ground glass opacities (Supplementary Fig. 2b). However, patient 2 developed rapidly progressive dyspnea with increasing NT-proBNP levels. Cardiac magnetic resonance (CMR) imaging showed right ventricle failure and signs of secondary remodeling as evidenced by the presence of late gadolinium enhancement (LGE) in the right ventricle. Pulmonary function test remained stable, and D-dimers did not increase substantially (Fig. 1b). Right heart catheterization demonstrated pulmonary hypertension with elevated precapillary and postcapillary pressure. Despite similar initial clinical responses, patient 2 demonstrated less pronounced decreases in anti-Jo1 autoantibody titers compared to patient 1 (Extended Data Fig. 2c). Eight months after baseline, CD19^+^ B cells reappeared in the blood, and the patient reported reoccurrence of mild mechanic’s hands and increase in CRP (Extended Data Fig. 2d) and myalgias despite normal CK levels. Immediate infusion of RTX resulted in depletion of peripheral B cells in the blood and improvement of clinical symptoms. Unlike the initial induction therapy with blinatumomab, the anti-Jo1 autoantibody titer showed no considerable reduction under maintenance therapy with RTX (Extended Data Fig. 2c). However, the patient maintained normal CK levels and remained off glucocorticoids throughout the observation period. TIS reached 60% after 3 months and 12 months.

Patient 3 (ASyS): Within 1 month after blinatumomab, CK levels normalized and 6MWD increased (650 m versus 442 m at baseline) (Fig. 1c). Myositis on MRI resolved (Supplementary Fig. 1c). Mechanic’s hands improved. After 6 months, muscle function improved further with increases in 6MWD and MMT8 scores (120 versus 52 at baseline). Due to an increase in peripheral B cells at month 5, along with stabilization of anti-Jo1 autoantibody titers, after a prior continuous decline (Extended Data Fig. 2e), RTX was administered. Patient 3 maintained glucocorticoid-free disease control and normal CRP levels throughout the follow-up period (Extended Data Fig. 2f). The TIS reached 88% after 11 months.

Patient 4 (ASyS): One month after treatment with blinatumomab, the previously bedridden patient started walking again with a walker. CK and CRP declined and normalized after 4 months (Fig. 1d and Extended Data Fig. 2g,h), and mechanic’s hands improved. A fracture of the patient’s right humerus occurred 3 months after blinatumomab and delayed the administration of RTX to day 150. In combination with previous amputation of the lower right leg and repetitive muscle biopsies of the patient’s left leg, it prevented meaningful assessments of muscle strength, 6MWD and TIS. Pulmonary function test improved (Fig. 1d), and HRCT after 4 months documented regressive consolidations (Supplementary Fig. 2c).

Patient 5 (ASyS): Already within the first 2 weeks of follow-up, the patient’s muscle function improved with increases in MMT8 from 118 at baseline to 144. The 6MWD increased, and CK and CRP levels declined (Fig. 1e and Extended Data Fig. 2i,j). FVC and DLCO remained stable (Fig. 1e). The TIS reached 75% after 2 months.

### Adverse events associated with blinatumomab in patients with ASyS

**Extended Data Fig. 3:**
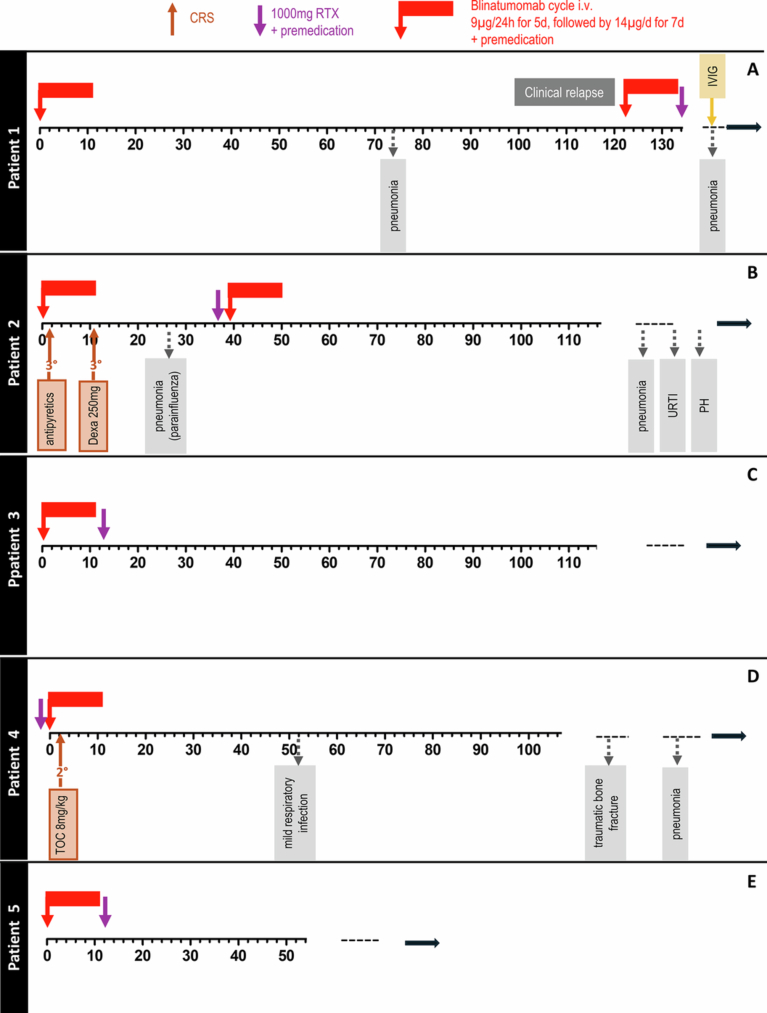
Schematic representation of adverse events in ASyS patients. **A**–**E**: Timeline of AEs (CRS, infection) and corresponding treatment after blinatumomab in patients 1 - 5 with ASyS (until end of follow-up). *Abbreviations*: AE, adverse events; CRS, cytokine release syndrome; RTX, rituximab; IVIG, intravenous immunoglobulin; Dexa, dexamethasone; URTI, upper respiratory tract infection; PH, pulmonary hypertension; TOC, tocilizumab. Rituximab premedication included: 1 g paracetamol p.o., 2 mg clemastine i.v. and 100 mg dexamethasone i.v.; Blinatumomab premedication included: 1 g paracetamol p.o., 2 mg clemastine i.v. and 20 mg dexamethasone i.v.

CRS occurred in patients 2 and 4. Patient 2 developed CRS grade 3 (fever and hypoxia) twice, on the first day and the last day of the first infusion cycle. The first episode of CRS subsided within less than 2 hours after pausing the infusion and administering paracetamol; the second CRS episode was more persistent and was treated with 250 mg of prednisolone. Patient 4 developed grade 2 CRS (hypotension) on the second day of blinatumomab and received tocilizumab. Patients 1 and 2 acquired infections that required antibiotic treatment. Patient 1 developed pneumonia with fever after reinduction with blinatumomab. All symptoms resolved within 5 days after empiric antibiotic treatment and supplementation with intravenous immunoglobulins (IVIG). Patient 2 presented with dyspnea and fever 2 weeks after the first cycle. The patient’s symptoms resolved within 4 days under antibiotic treatment. During follow-up, he developed three respiratory infections, two of which were treated with antibiotics. Patient 4 reported a mild respiratory infection 4 weeks after blinatumomab that subsided without treatment. The patient also developed pneumonia 5 months after initiation with blinatumomab with evidence of herpes simplex virus in the bronchoalveolar lavage, which was treated with aciclovir for 9 days and antibiotics (due to suspected bacterial superinfection). Patients 3 and 5 did not report any infections (Extended Data Fig. 3 and Extended Data Table 1).

### Cellular and serological changes in patients with ASyS

**Fig. 2:**
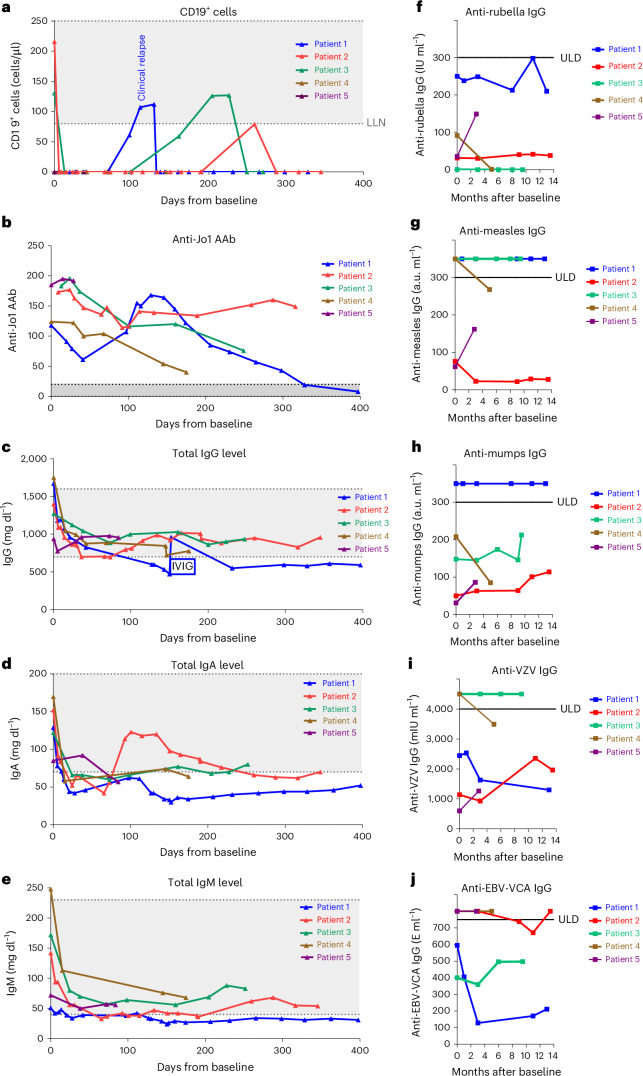
Immunological outcomes in patients with ASyS in response to induction therapy with blinatumomab and maintenance therapy with RTX. **a**, Changes of CD19^+^ B cells in patients 1−5. **b**, Levels of anti-Jo1 autoantibodies. **c**–**e**, Levels of total IgG, IgA and IgM. **f**–**j**, Chances of antiviral antibodies induced by vaccination or infection. Anti-Jo1 AAb, anti-Jo1 autoantibody (measured by ELISA); IgA, immunoglobulin A (normal range: 70−200 mg dl^−1^); IgG, immunoglobulin G (normal range: 700−1,600 mg dl^−1^); IgM, immunoglobulin M (normal range: 40−230 mg dl^−1^); VCA, viral capsid antigen.

Peripheral B cells were rapidly and completely depleted in all patients with ASyS after treatment with blinatumomab (Fig. 2a). Complete repression of peripheral B cells was maintained in patients 4 and 5, whereas patients 1, 2 and 3 had one event with reoccurrence of peripheral B cells each. This was associated with clinical relapse in patient 1, which triggered reinduction therapy with blinatumomab. The other patients did not demonstrate clinical symptoms and were managed with RTX only.

The titers of anti-Jo1 autoantibodies initially decreased in all patients with ASyS after induction therapy with blinatumomab (Fig. 2b). However, the magnitude of the decline and the kinetics during follow-up were variable across individual patients, which might reflect, in part, differences in the maintenance regime. In patient 1, the anti-Jo1 autoantibody titers increased with the reoccurrence of B cells during her flare. After reinduction therapy with blinatumomab and with tight B cell depletion with repetitive RTX infusions, the anti-Jo1 autoantibody titers progressively declined and fell below the upper limit of normal (ULN) after 12 months (Fig. 2b). Patient 2 demonstrated milder decreases in anti-Jo1 autoantibody titers, which reached a plateau after 4 months. In patients 3 and 4, the anti-Jo1 autoantibody titer decreased progressively after blinatumomab. Due to the short follow-up period, the kinetics of the anti-Jo1 autoantibodies in patient 5 remain inconclusive. Changes in anti-Jo1 autoantibody titers did not, thus, consistently correlate with clinical responses, and glucocorticoid-free disease control was also achieved in patients who maintained detectable levels of anti-Jo1 autoantibodies.

The total IgG levels decreased in patients with ASyS but remained within the lower normal range in all patients except patient 1 (Fig. 2c). Patient 1 was substituted with IVIG 20 g intravenously once in the setting of hypogammaglobulinemia-associated pneumonia. IgA and IgM levels declined initially but remained stable on a low normal or slightly subnormal level (Fig. 2d,e).

The levels of protective antibodies, such as anti-rubella IgG, anti-mumps IgG, anti-measles IgG, anti-varicella zoster virus (VZV) IgG or anti-Epstein−Barr virus (EBV) IgG, did not substantially change in all patients treated with blinatumomab (Fig. 2f–j).

The ongoing chronic infection with *Mycobacterium kansasii* in patient 3 progressively healed under antibiotic therapy initiated 2 weeks before the first blinatumomab infusion (Supplementary Fig. 3). Infections with hepatitis B (in patients 1 and 4) or *Mycobacterium tuberculosis* (in patient 1) did not reactivate.

### Histological changes in muscle biopsies in patients with ASyS

**Extended Data Fig. 4:**
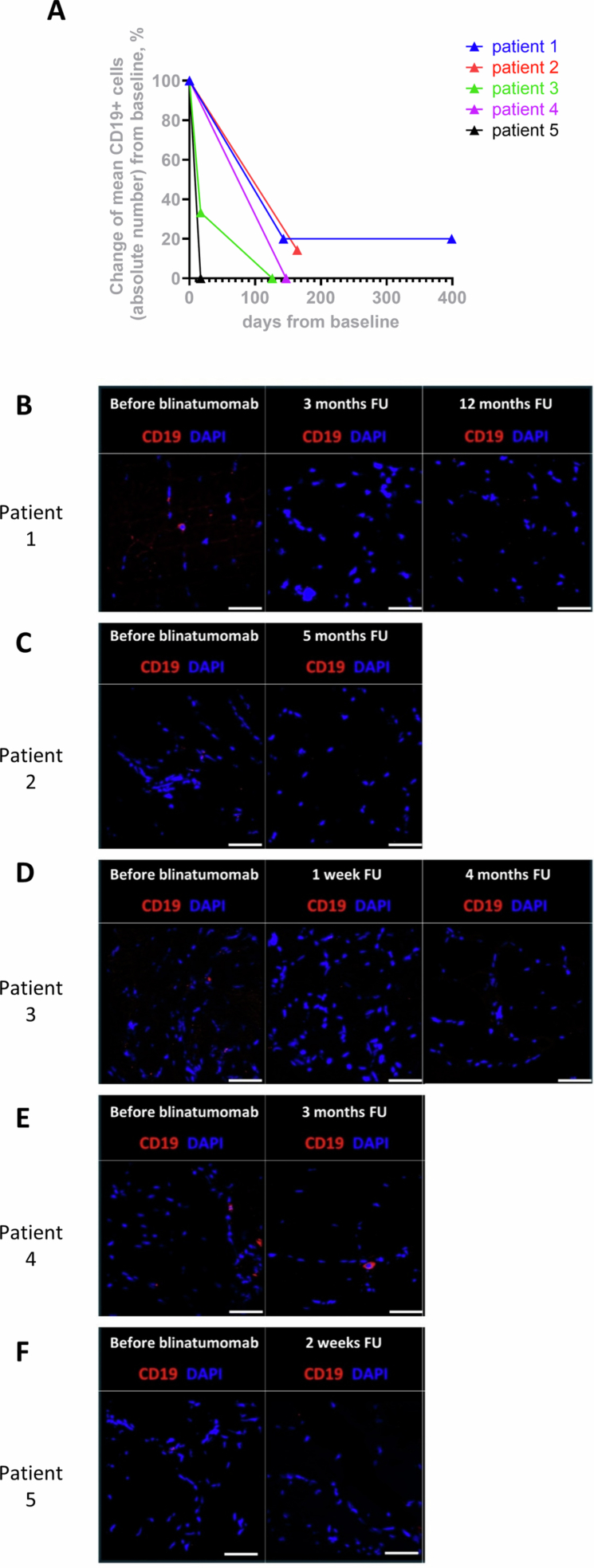
Changes in the number of CD19+ cells in the skeletal muscle biopsies of patients with ASyS in response to treatment with blinatumomab/RTX. **A**: Quantification of CD19+ cells in muscle cryosections of patients 1 - 5, before and after treatment with blinatumomab. **B**–**F**: Representative immunofluorescence images of CD19 (red). Nuclei are counterstained with DAPI in blue. Scale bars represent 50 µm. *Abbreviations*: DAPI, 4′,6-diamidino-2-phenylindole.

Treatment with blinatumomab strongly decreased the numbers of CD19^+^ B cells in all muscle biopsies, with complete depletion in patients 3, 4 and 5 and only minimal B cell counts in patients 1 and 2 (Extended Data Fig. 4).

**Extended Data Fig. 5:**
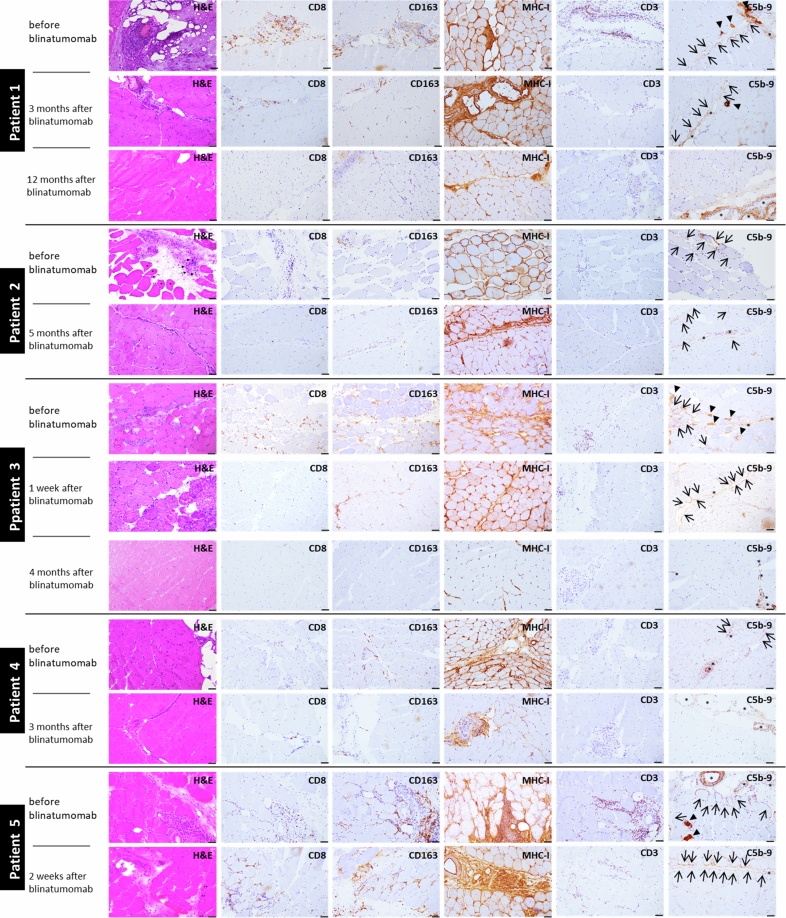
Histological changes in skeletal muscle biopsies of patients with ASyS after treatment with blinatumomab. Muscle biopsies of patients 1 - 5 before and after blinatumomab. M. quadriceps femoris was biopsied in patients 1, 3, 4, and 5. In patient 2, M. biceps brachii was biopsied. H&E staining and immunohistochemistry staining with anti-CD8, CD163, MHC-I, CD3 or C5b-9 and counterstaining with hemalum are shown at magnification x200. Scale bars = 50 µm. *Abbreviations*: H&E, Hematoxylin-Eosin. Arrows indicate pathological, sarcolemmal perifascicular and arrowheads pathological sarcoplasmic complement deposits, respectively, while asterisks indicate physiological complement expression on medium-sized vessels. Double asterisks (H&E staining) are indicative for regenerating muscle fibers.

In patient 1, a first follow-up biopsy 3 months after induction therapy with blinatumomab demonstrated reduced numbers of necrotic muscle fibers, regression of inflammatory infiltrates, modestly decreased major histocompatibility complex class I (MHC-I) expression and evidence of regeneration. On 12-month follow-up, necrotizing muscle fibers were no longer detectable, and only scattered CD8^+^ T cells and few macrophages and histiocytes were remaining. MHC I expression became limited to the sarcolemma of some muscle fibers, in addition to physiological expression on endomysial capillaries. Complement deposits were only present in a physiological manner in medium-sized arterioles as compared to sarcoplasmic and sarcolemmal detection at baseline (Extended Data Fig. 5).

In patient 2, infiltrating CD4^+^ T cells were absent after 5 months, and only small numbers of CD8^+^ T cells and macrophages were detected. MHC class I expression became restricted to focal areas. In addition to physiological complement deposits on medium-sized arterioles, only weak complement deposits were present on some muscle fibers, in contrast to strong sarcolemmal expression at baseline (Extended Data Fig. 5).

In patient 3, a muscle biopsy 1 week after the initiation of blinatumomab demonstrated rapid depletion of CD19^+^ B cells by blinatumomab (Extended Data Fig. 4d). Another biopsy after 4 months showed persistent depletion of CD19^+^ B cells, complete absence of previous necrotic changes and CD4^+^ cells and reduced CD8^+^ cell counts. MHC-I expression decreased upon treatment, with only physiological MHC-I expression on endomysial capillaries remaining after 4 months. Fiber necrosis was no longer detectable upon treatment, and complement deposits were only present physiologically on medium-sized arterioles after 4 months (Extended Data Fig. 5).

In patient 4, a follow-up biopsy after 3 months demonstrated decreased CD8^+^ cell counts, decreased expression levels of MHC-I antigen detectable only in few focal areas and absence of pathological complement deposits outside medium-sized arterioles (Extended Data Fig. 5).

The baseline muscle biopsy of patient 5 demonstrated strong immune cell infiltration, pathologic complement deposition, increased MHC-I expression and extensive muscle fiber necrosis. Two weeks after blinatumomab, immune cell infiltration decreased, with most pronounced changes in macrophage and histocyte counts. The upregulation of MHC-I began to decline. Sarcoplasmic complement deposits were no longer detectable, and the sarcolemmal complement deposits decreased. In addition, signs of regeneration appeared (Extended Data Fig. 5).

### Outcome of patients with SSc receiving induction therapy with teclistamab and maintenance therapy with RTX

**Fig. 3:**
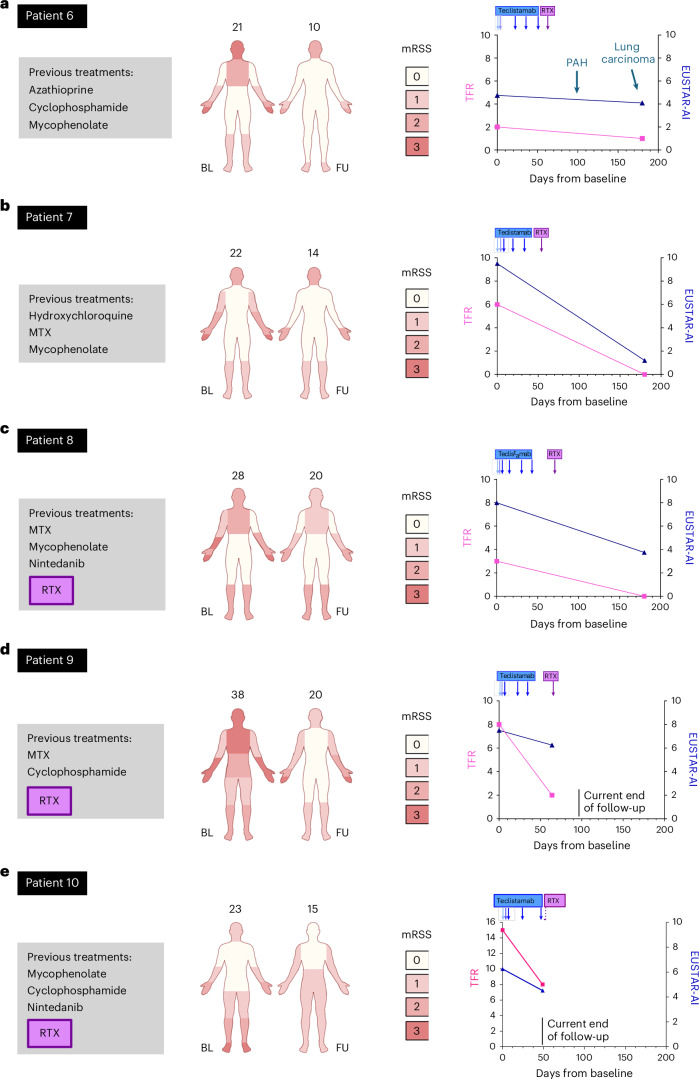
Clinical outcomes of individual patients with SSc in response to induction therapy with teclistamab and maintenance therapy with RTX. **a**–**e**, Treatment history and changes in mRSS, TFR and EUSTAR-AI before and after induction therapy with teclistamab and maintenance therapy with RTX (at the end of follow-up) in patients 6–10 with SSc. BL, baseline; FU, follow-up.

**Extended Data Fig. 6:**
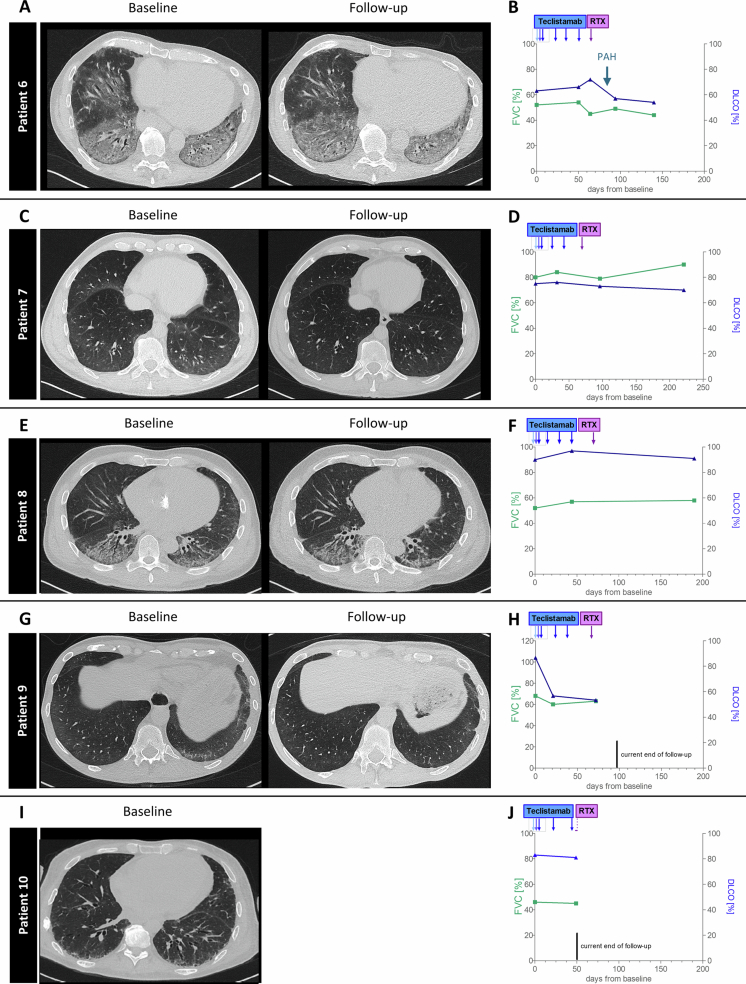
Changes of the mRSS in individual patients with SSc over time after induction therapy with teclistamab and maintenance therapy with RTX. Changes in HRCT and lung function of patients with SSc with interstitial lung disease before and after treatment with teclistamab and RTX. **A**, **C**, **E**, **G**: Comparison of HRCT findings before and after teclistamab/RTX in patients 6-9, respectively. **I** HRCT of patient 10 before teclistamab/RTX. **B**, **D**, **F**, **H**, **J**: Changes of FVC (percent predicted) and DLCO_VA (DLCO, percent predicted). *Abbreviations*: HRCT, High-Resolution computed tomography; FVC, forced vital capacity; DLCO, diffusing capacity of the lung for carbon monoxide; RTX, rituximab. **A**–**E**: Treatment history and changes in mRSS after teclistamab and RTX in patients 6 – 10. *Abbreviations*: mRSS, modified Rodnan skin score; RTX, Rituximab.

Patient 6 (SSc): Upon induction therapy with teclistamab, modified Rodnan skin score (mRSS) improved from 21 to 10 within 6 months (Fig. 3a and Supplementary Fig. 4a). FVC declined from 53% at baseline to 47% at month 5, and DLCO stabilized (52% after 5 months versus 48% at baseline) (Extended Data Fig. 6a,b). The indices of ground glass opacities (GGOI) and reticular changes (fibrosis index (FIBI)) on HRCT, quantified as described previously^19^, remained stable (Supplementary Fig. 5). CMR imaging showed normal cardiac function with a slight deterioration of left ventricular ejection fraction (LV-EF), T1 times and extracellular volume (ECV) (Supplementary Figs. 7 and 8). The European Scleroderma Trials and Research Group Activity Index (EUSTAR-AI) decreased from 4.75 to 4.09 (Fig. 3a).

**Extended Data Fig. 7:**
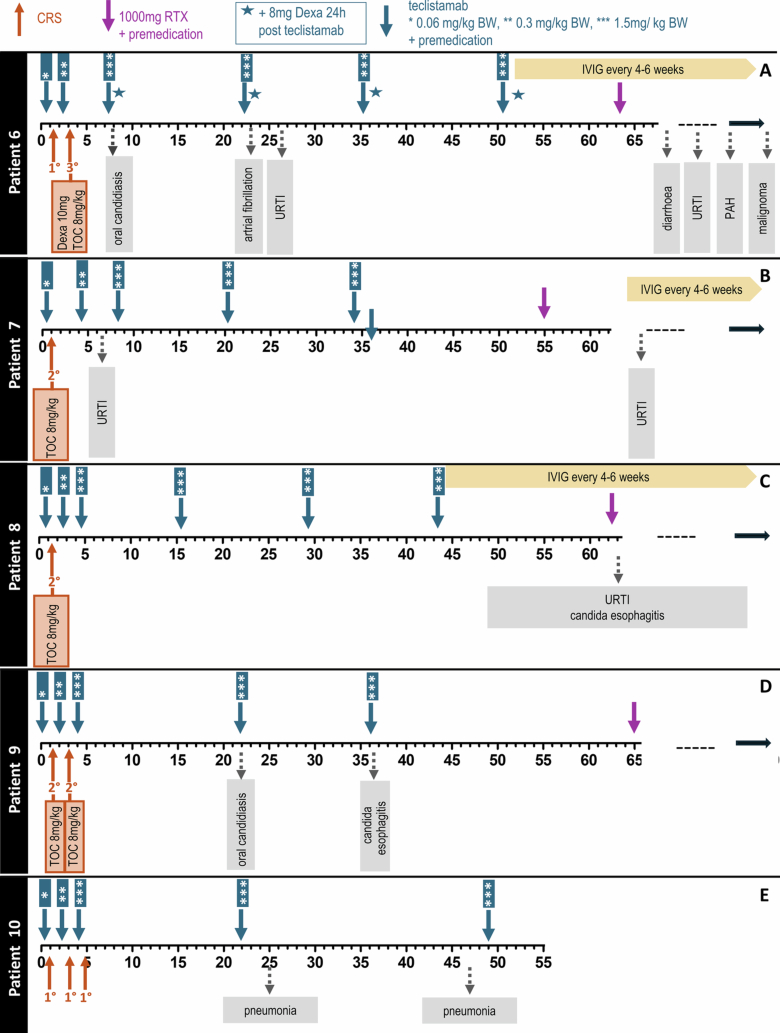
Schematic representation of adverse events in SSc patients treated with teclistamab/RTX. **A**–**E**: Timeline of AEs (CRS, infection) and corresponding treatment after teclistamab and RTX in patients 6 - 10 with SSc. *Abbreviations*: AE, adverse events; CRS, cytokine release syndrome; RTX, Rituximab; IVIG, intravenous immunoglobulin; Dexa, dexamethasone; URTI, upper respiratory tract infection; PAH, pulmonary arterial hypertension; TOC, tocilizumab. Rituximab premedication included: 1 g paracetamol p.o., 2 mg clemastine i.v. and 100 mg dexamethasone i.v.; Teclistamab premedication included: 1 g paracetamol p.o., 2 mg clemastine i.v. and 16 mg dexamethasone i.v.

The patient developed a relapse of atrial fibrillation after 1 month. He also experienced progressive worsening of dyspnea after an initial improvement (from 9/10 at baseline to 6/10 at month 2 to 8/10 at month 5) (Extended Data Fig. 7). Right heart catheterization established the diagnosis of pulmonary hypertension with elevated precapillary pressure. Moreover, a large cell neuroendocrine pulmonary carcinoma was diagnosed after 7 months. Pulmonary hypertension and the carcinoma complicate the interpretation of the treatment effects of teclistamab as they may have negatively impacted FVC, DLCO, ejection fraction and EUSTAR-AI.

Patient 7 (SSc): The patient’s mRSS improved from 22 to 14 within 6 months after the first teclistamab infusion (Fig. 3b and Supplementary Fig. 4b). All tendon friction rubs (TFRs) resolved (Fig. 3b). FVC improved (90% versus 80% at baseline), and DLCO remained stable (70% versus 76% at baseline) after 6 months (Extended Data Fig. 6c,d). HRCT-based parameters of ILD modestly regressed after 3 months (Supplementary Fig. 5). The patient’s SSc with primary heart involvement (SSc-pHI) improved as evidenced by declining high-sensitivity cardiac troponin T (hsTnT) levels and reduced T1 times and ECV on CMR imaging (Supplementary Figs. 7 and 8). He regained 15 kg of body weight. The EUSTAR-AI decreased from 9.5 to 1.2 (Fig. 3b).

Patient 8 (SSc): The patient’s mRSS decreased from 28 to 20 (Fig. 3c and Supplementary Fig. 4c). All TFRs resolved (Fig. 3c). ILD stabilized with slightly improved FVC (58% versus 52% at baseline), improved FIBI and stable DLCO and GGOI (Extended Data Fig. 6e,f and Supplementary Fig. 5). Levels of hsTnT declined. However, the follow-up CMR imaging at month 7 showed increased T1 values, pathologic ECV, persisting LGE and a subclinical decline in LV-EF (Supplementary Figs. 7 and 8). Echocardiography confirmed a trend toward decreasing LV-EF, although the LV-EF remained still within the normal range. He regained 2.5 kg of body weight, and his EUSTAR-AI decreased from 8 to 3.75 (Fig. 3c).

Patient 9 (SSc): The patient’s mRSS improved from 38 to 20 within 2 months (Fig. 3d and Supplementary Fig. 4d). All TFRs regressed and most resolved (Fig. 3d). During the short follow-up period of 3 months, his FVC remained stable; his DLCO declined directly after induction therapy (87% versus 104% at baseline) but remained stable on further follow-up (Extended Data Fig. 6g,h). HRCT demonstrated stable ground glass opacities (Extended Data Fig. 6d). Echocardiography showed normalized left and right ventricular ejection fractions. Values decreased for hsTnT (54ng l^−1^ versus 81 ng l^−1^ at baseline) and NT-proBNP (909 pg ml^−1^ versus 1,763 pg ml^−1^ at baseline). Muscle MRI showed regression of edema (Supplementary Fig. 6).

Patient 10 (SSc): Already within 1 month, the patient’s mRSS (15 versus 23 at baseline) and the number of TFRs (Fig. 3e and Supplementary Fig. 4e) decreased. FVC and DLCO remained stable (Extended Data Fig. 6i,j). Values for hsTnT and NT-proBNP also declined. CK values normalized (106 U l^−1^ versus 741 U l^−1^ at baseline), and arthritis improved.

### Adverse events associated with teclistamab treatment in patients with SSc

CRS occurred more frequently in patients with SSc treated with teclistamab than in patients with ASyS treated with blinatumomab (Extended Data Figs. 3 and 7 and Extended Data Table 1). All patients with SSc experienced CRS, which occurred most within 48 hours after the first dose (grade 1 CRS in patients 6 and 10 and grade 2 CRS in patients 7, 8 and 9, treated with tocilizumab 8 mg kg^−1^ body weight) (Extended Data Fig. 7). Patients 6, 9 and 10 developed additional CRS at later timepoints. Patient 6 developed grade 3 CRS after the second teclistamab dose, which was treated with tocilizumab and 8 mg of dexamethasone and required short-term oxygen therapy as well as short-term vasopressor therapy. After this CRS, we added prophylactic treatment with 8 mg of dexamethasone in patient 6 before further teclistamab applications. Patient 9 developed grade 2 CRS after the second teclistamab dose, which was treated with tocilizumab. Patient 10 showed grade 1 CRS after the second and third dose of teclistamab (Extended Data Fig. 7 and Extended Data Table 1). In all patients, CRS symptoms completely resolved within the same day.

No ICANS occurred.

Episodes of seasonal respiratory infection occurred in all patients with SSc. Patients 6, 8, 9 and 10 developed oral and/or esophageal candidiasis, which was treated with amphotericin B suspension and fluconazole. Patients 7 and 8 had recurrent episodes of cough, which were intermittently treated with oral antibiotics. Patient 10 developed two episodes of pneumonia after the fourth administration of teclistamab, which were treated with intravenous antibiotics. Patient 6 developed chronic mild diarrhea.

### Serological changes in patients with SSc

**Fig. 4:**
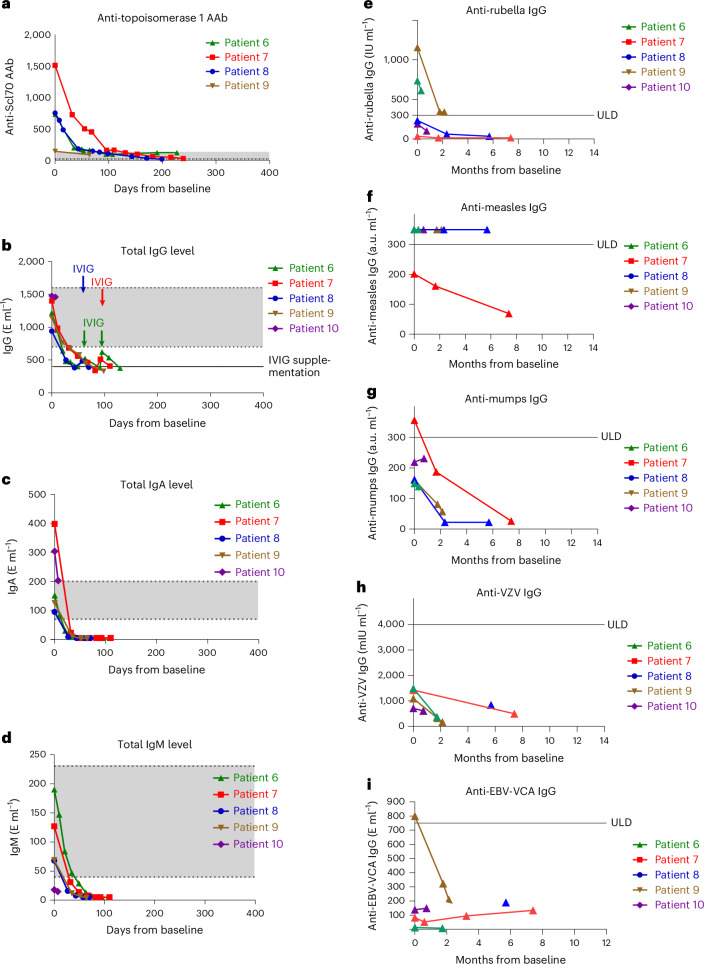
Immunological outcomes in patients with SSc in response to induction therapy with teclistamab and maintenance therapy with RTX. **a**, Changes of anti-topoisomerase 1 autoantibody levels in patients 6−10. **b**–**d**, Serum levels of total IgG, IgA and IgM. **e**–**i**, Changes of antiviral antibodies induced by vaccination or infection. AAb, autoantibody; IgA, immunoglobulin A (normal range: 70−400 mg dl^−1^); IgG, immunglobulin G (normal range: 700−1,600 mg dl^−1^); IgM, immunoglobulin M (normal range: 40−230 mg dl^−1^); ULD, upper limit of detection; VCA, viral capsid antigen.

**Extended Data Fig. 8:**
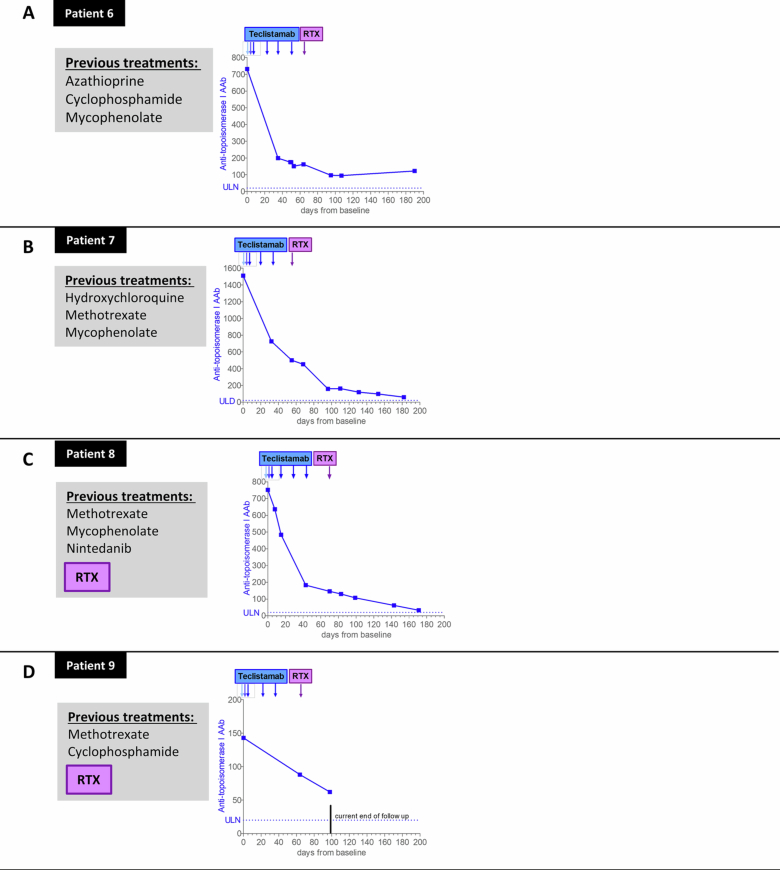
Changes in anti-topoisomerase I autoantibody levels in response to treatment with teclistamab and RTX. **A**–**D**: Individual outcomes in patients 6 – 9. *Abbreviations*: RTX, Rituximab.

Anti-topoisomerase autoantibodies declined in all patients with SSc upon treatment with teclistamab (Fig. 4a and Extended Data Fig. 8). In patients 7 and 8, titers decreased progressively and fell underneath the ULN at the end of the follow-up period. In patient 6, the titers declined rapidly initially but reached a plateau at 17% of the baseline titers after 3 months (shortly before the diagnosis of his carcinoma). Despite limited follow-up time, the anti-topoisomerase 1 autoantibody titers already started to decrease in patient 9. For patient 10 with anti-fibrillarin autoantibodies, quantification of autoantibody levels was not performed. Serum IgA and IgM levels dropped below the detection limit in patients 6−9 (Fig. 4c,d). IgG dropped below 400 mg dl^−1^ by day 49 (Fig. 4b). In contrast to the observations with blinatumomab in patients with ASyS, vaccination-induced IgG titers also declined in patients with SSc treated with teclistamab (Fig. 4e–g).

Consistent with previous reports^20^, teclistamab induced depletion of circulating B cells already before maintenance therapy with RTX. CD19^+^ B cells had already been depleted from the peripheral blood of patients 9 and 10 at the time of teclistamab induction due to previous RTX therapy.

### Depletion of B cells in the skin of patients with SSc

**Extended Data Fig. 9:**
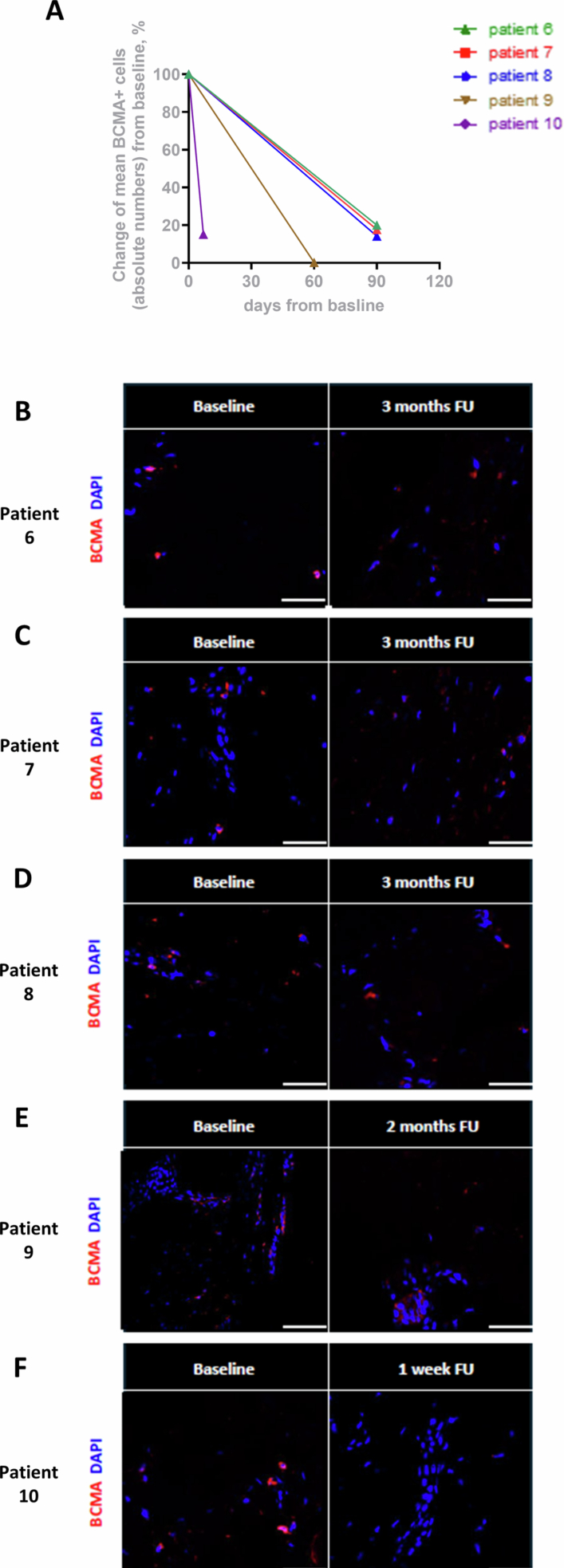
Changes in the number of BCMA+ cells in the skin of SSc patients in response to treatment with teclistamab/RTX. **A**: Quantification of BCMA+ cells in the skin of patients 6 - 10, before and after treatment with teclistamab. **B**–**F**: Representative immunofluorescence images of BCMA (red) in the skin of patients 6 - 10 before and after treatment with teclistamab/RTX. Nuclei are counterstained with DAPI and represented in blue. Scale bars represent 50 µm. DAPI, 4′,6-diamidino-2-phenylindole.

Analyses of skin biopsies collected at baseline and after teclistamab therapy by immunofluorescence and immunohistochemistry staining demonstrated strong decreases in the numbers of BCMA^+^ cells by teclistamab in all five patients with SSc (Extended Data Fig. 9 and Supplementary Fig. 9). Confirmatory experiments using co-detection by indexing (CODEX) in patients 7, 8 and 9 using additional B cell and plasma cell markers (CD19, CD20, CD38 and CD138) confirmed the decrease of plasma cells upon teclistamab treatment (Supplementary Fig. 10).

## Discussion

We report safety and efficacy of blinatumomab and teclistamab in patients with ASyS and SSc, respectively. Induction therapy with both TCEs demonstrated clinical efficacy and enhanced disease control, despite discontinuation of all other immunosuppressive and antifibrotic drugs. Induction therapy with blinatumomab in patients with ASyS normalized CK levels, improved MMT8 scores, increased the 6MWD and stabilized lung function parameters in patients with ILD. Induction therapy with teclistamab induced regression of skin fibrosis, resolution of TFRs, stabilization of SSc-associated interstitial lung disease (SSc-ILD) and improvement of the EUSTAR-AI in patients with SSc. These findings are consistent with a recent case report on efficacy of teclistamab in a patient with SSc with relapse after hematopoietic stem cell transplantation (HSCT)^31^. Although the increased sensitivity of patients with SSc to immunomodulatory treatment after HSCT may have facilitated the response in this single patient^32^, our results indicate that teclistamab might be effective in a broader SSc population.

Interpretation of the clinical efficacy needs to consider that all patients had difficult-to-treat, treatment-refractory disease. All patients in the ASyS cohort and in the SSc cohort previously did not achieve disease control with at least three different treatments. Of note, all patients with ASyS and three out of five patients with SSc previously did not respond to therapy with RTX. Blinatumomab and teclistamab strongly decreased the numbers of CD19-expressing and BCMA-expressing target cells in affected muscle and skin tissues, respectively, as demonstrated by consecutive assessments of tissue biopsies. By contrast, RTX is known to have limited cytotoxic efficacy in tissue with persistence of CD20^+^ cells^33^. This effective depletion in tissues by blinatumomab and teclistamab together with targeting of additional, disease-relevant B cell populations may explain the efficacy of both TCEs as induction therapy despite prior RTX failure.

Most patients of our cohort had already advanced disease with major organ damage. Myocardial involvement and ILD rendered those patients ineligible for high-dose chemotherapy followed by HSCT as another salvage therapy. Patients 2 and 6 would also be excluded from currently ongoing CAR-T cell trials. TCE may, thus, provide a treatment option for patients with ASyS and SSc with advanced disease, in whom cellular therapies are currently not used.

After treatment with blinatumomab and teclistamab, decreases in autoantibody titers were observed in all patients. Both TCEs decreased autoantibody titers also in patients with absent circulating B cells due to previous RTX therapy. The reduction in autoantibody titers was more pronounced in patients with SSc treated with teclistamab than in patients with ASyS treated with blinatumomab. This reflects the broader B cell depletion by teclistamab with additional targeting of CD19^−^ long-lived plasma cells as a major antibody-producing cell population that is not affected by blinatumomab. However, the more stringent application of maintenance therapy with RTX in the SSc group compared to the ASyS group may have contributed. The clinical response to blinatumomab in all patients despite persistence of anti-Jo1 autoantibodies provides evidence that the therapeutic effect is mediated not only by reduction of autoantibody titers but also by inhibition of other disease-promoting functions of B cells, such as cytokine production and antigen presentation.

Given the limited half-life of both TCEs, maintenance therapy with RTX was initiated aiming to prevent replenishment of tissue niches with pathogenic B cells. As B cells recurring after TCE treatments are mostly naive cells that express CD20, they can be targeted by RTX^34^. Thus, application of RTX initiated during phases of TCE-induced depletion can prevent redifferentiation of plasmablasts and plasma cells from earlier B cell precursors. However, effective prevention of replenishment requires stable depletion of B cells during the maintenance phase. Unless all autoantibody-producing clones are completely eradicated, pathogenic B cell precursors can redifferentiate and repopulate B cell niches during intervals without B cell depletion (for example, patient 1). Our empirical approach in SSc with application of RTX 2 weeks after the last teclistamab dose resulted in effective depletion of peripheral B cells throughout the observation period, and none of the patients with SSc demonstrated evidence of disease activity during follow-up. However, our initial approaches in the first patients with ASyS did not result in persistent suppression of B cell counts. Circulating B cells reoccurred in patients 1, 2 and 3, which was associated with reappearance of disease activity in patients 1 and 2. Patient 3 remained in clinical remission despite increasing peripheral B cell counts. Thus, although all patients responded to initiation therapy with TCEs, insufficient B cell depletion during follow-up was associated with a high risk of relapse. This suggests that the dosing schedules for RTX should aim for persistent, complete suppression of B cells. Consequently, the RTX intervals cannot be guided by peripheral B cell counts or autoantibody levels but need to be determined empirically in follow-up studies. Moreover, the potential role of other B-cell-targeting therapies needs to be explored.

Patients treated with blinatumomab demonstrated lower rates of infections as compared to patients treated with teclistamab, despite repetitive IVIG supplementation in the telistamab cohort. Four out of five patients treated with blinatumomab maintained normal levels of immunoglobulins, the immunoglobulin levels dropped below 400 mg dl^−^^1^ in all patients treated with teclistamab during longer follow-up^34,35^. The lower infection rate may result from less pronounced effects of blinatumomab on immunoglobulin levels. Although four out of five patients treated with blinatumomab maintained normal levels of immunoglobulins, the immunoglobulin levels dropped below 400 mg dl^−1^ in all patients with SSc with longer follow-up. Vaccination-induced immunoglobulin titers remained stable in patients treated with blinatumomab, whereas they declined in patients treated with teclistamab. In the context of risk of infections, it is also important to note that the preexisting *M. kansasii*-induced ulcers in patient 3 healed progressively and that hepatitis B and tuberculosis infections in patient 1 did not reactivate under blinatumomab induction therapy and RTX maintenance therapy.

CRS was also less common in blinatumomab-treated patients as compared to teclistamab-treated patients, with three CRS events in the blinatumomab cohort as compared to nine CRS events in the teclistamab cohort.

Beyond CRS and infectious complications, we noticed additional severe adverse events that have not been associated with TCE treatment so far^34–36^. Patients 2 and 6 were newly diagnosed with pulmonary hypertension during follow-up. Moreover, a large cell neuroendocrine pulmonary carcinoma was diagnosed in patient 6. For the case of the pulmonary carcinoma, a retrospective analysis of the baseline HRCT indicated that unspecific signs may have already been present before TCE treatment. Pulmonary hypertension may also have been present at mild stage before TCE treatment. Induction of pulmonary hypertension by TCE would be unexpected as RTX has been reported to be safe in patients with SSc with established pulmonary arterial hypertension^36,37^. However, careful monitoring is warranted, and in-depth screening for pulmonary hypertension should be considered for future patients before initiation of TCE therapy.

Individual patients did not only show major differences in the rate and severity of adverse events but also in treatment response of cardiac manifestations. Although hsTnT decreased in all patients with SSc, only patient 7 demonstrated improvement in T1 and ECV on CMR imaging, whereas these parameters rather worsened in patients 6 and 8. Although the worsening might be explained by the newly diagnosed pulmonary hypertension in patient 6, these findings are indicative of progressive fibrotic remodeling of the heart in patient 8. This heterogenous response warrants close monitoring of patients with SSc with preexisting myocardial disease under teclistamab therapy.

Our findings have limitations. These include the relatively small number of treated patients, the short follow-up period for some of these patients and the fact that patients were treated with TCEs as part of a compassionate use program and not in a clinical trial, which limits comparability between the individual patients, prohibits a direct comparison of induction therapy with TCEs versus standard of care and does not allow the comparison of different regimens for maintenance therapy. Follow-up studies are also required to determine the optimal dosing schedules and regimen for maintenance therapy; other B-cell-targeting therapies than RTX or conventional disease-modifying antirheumatic drugs (DMARDs) such as mycophenolate mofetil (MMF) may also offer potential to maintain disease control after induction therapy with TCEs.

In summary, we provide evidence that induction therapy with blinatumomab or teclistamab followed by maintenance therapy with RTX is effective in the treatment of patients with severe, treatment-refractory ASyS or SSc, respectively. Further studies with additional patients and longer follow-up are required to confirm these findings, to establish optimal protocols for maintenance therapy and to identify patients with optimal benefit−risk profiles.

## Methods

### Patient characteristics at baseline

All patients with ASyS and SSc were treated at the University Hospital of Düsseldorf after written informed consent according to CARE guidelines and in compliance with Declaration of Helsinki principles. Sampling of patient material was performed, and ethical approval for our biobank was obtained (2022-2189 and 2023-2561). Treatment was conducted as part of a compassionate use program without a specific research protocol.

All patients with ASyS were positive for anti-Jo1 autoantibodies. Three out of five patients also showed autoantibodies against Ro-52. Each patient with ASyS previously did not respond to at least four immunomodulatory medications, including RTX. Detailed characteristics of patients with ASyS at baseline are shown in Table 1.

Patient 1 (ASyS) was a 65-year-old woman diagnosed with anti-Jo1^+^ ASyS in 2022. She previously experienced an infection with hepatitis B. Initial symptoms included muscle weakness in the legs, neck and shoulders, dyspnea, mechanic’s hands and weight loss. Laboratory tests showed elevated CK, and MRI revealed severe myositis of both quadriceps muscles. She did not achieve the desired treatment response after treatment with methotrexate (MTX), azathioprine, high-dose IVIG and RTX (last infusion 2 months before baseline; B cells depleted in peripheral blood), with repetitive flares that required multiple pulses of prednisolone. She developed ILD on HRCT with a non-specific interstitial pneumonia (NSIP) pattern and declining FVC and diffusing lung capacity for carbon monoxide-alveolar volume (DLCO-VA).

Patient 2 (ASyS) was a 36-year-old man diagnosed with anti-Jo1^+^ ASyS in 2022, after initial diagnosis of seronegative rheumatoid arthritis in 2021, which had been treated with MTX and certolizumab. At the time of diagnosis of ASyS, he presented with muscle weakness in arms and shoulders with elevated CK levels, mechanic’s hands and recurrent arthritis of hands and knees. Treatment with RTX was initiated in 2022. During treatment with RTX, he developed ILD with an NSIP pattern and myocardial involvement as evidenced by CMR imaging with increased native global T1 times and ECV. Despite treatment with cyclophosphamide followed by MMF and tacrolimus, the ILD progressed rapidly, requiring oxygen supplementation. Tacrolimus was stopped and nintedanib initiated. Subsequently, flares of arthritis required repetitive pulses of prednisolone. At first presentation in our department in October 2023, he demonstrated severe and progressive dyspnea (NYHA III/IV). We replaced MMF with tofacitinib, resulting in better control of arthritis but not of myositis, pulmonary and cardiac involvement, despite additional uptitration of prednisolone.

Patient 3 (ASyS) was a 47-year-old man diagnosed with anti-Jo1^+^ ASyS in 2020. Symptoms included bilateral quadriceps weakness, elevated CK levels and mechanic’s hands. His CMR imaging did not demonstrate evidence of myocardial involvement at that time. He exhibited persistent serological activity and progressive muscle weakness despite treatment with MTX, azathioprine, high-dose IVIG and RTX and repeated courses of high doses of prednisolone. In 2024, he presented at our department with highly active myositis with necrosis on histology. He was unable to work and required assistance with activities of daily living. He demonstrated no signs of ILD on HRCT. On follow-up, CK levels increased further. He also presented with an active, enlarging ulcer on his lower right leg. Polymerase chain reaction testing revealed positivity for *M. kansasii*, and a triple antibiotic treatment was initiated that induced gradual healing.

Patient 4 (ASyS) was a 48-year-old woman diagnosed with anti-Jo1^+^ ASyS in 2020, after an initial, external diagnosis of psoriatic arthritis, which was treated sequentially with leflunomide, hydroxychloroquine, ixekizumab and apremilast. At the time of diagnosis of ASyS, MRI scans showed generalized myositis of all thigh muscle groups and myocarditis. ILD was detected in 2022. Treatment with MTX, IVIG, MMF, cyclophosphamide, ciclosporin and RTX (last infusion 1 week before baseline) failed to provide sufficient disease control, with recurrent flares of myositis, mechanic’s hands and progressive dyspnea. During these therapies, she developed pneumocystis jirovecii-induced acute respiratory distress syndrome (ARDS) requiring invasive ventilation, cardiac decompensation and severe deep vein thrombosis, leading to amputation of the right leg just below the knee. At first presentation in our department in 2025, HRCT demonstrated ILD with an NSIP pattern. Her CMR imaging showed increased native T1 values. Her FVC progressively declined, her demand for supplementary oxygen increased and she showed signs of active myositis on MRI and muscle biopsy.

Patient 5 (ASyS) was a 50-year-old woman with anti-Jo1^+^ ASyS diagnosed in 2020. Her initial symptoms included myalgia, arthritis and skin rash. ILD was detected in 2021. She experienced repetitive flares under treatment with azathioprine, MTX and hydroxychloroquine, requiring repetitive cycles of glucocorticoid pulses. After referral to our department in 2023, treatments with MMF, RTX (last infusion 3 months before baseline) and IVIG did not provide sufficient disease control, with repetitive flares of myositis, skin rash and progressive dependency on assistance to manage daily activities. She developed severe pneumonia with ARDS, requiring invasive ventilation and veno-venous extracorporeal membrane oxygenation in June 2024. Subsequent MRI and muscle biopsy demonstrated active myositis, and her HRCT showed progression of ILD with further declines of FVC.

All patients with SSc were diagnosed with dcSSc. Four patients were positive for autoantibodies against topoisomerase 1, and one patient was positive for anti-fibrillarin autoantibodies (patient 10). All patients demonstrated widespread skin fibrosis, ILD and SSc with primary heart involvement (SSc-pHI). Each patient failed previously to respond to at least three different immunmodulatory/antifibrotic drugs (Table 2).

Patient 6 (SSc) was a 65-year-old man diagnosed with dcSSc in 2006. He developed progressive ILD with declining FVC and DLCO despite treatment with cyclophosphamide, azathioprine and MMF. He was admitted to our department in May 2024 with an mRSS of 21, TFRs, an FVC of 52% and dyspnea NYHA III. CMR imaging demonstrated globally increased native T1 values and ECV according to local reference standards, consistent with SSc-pHI. His hsTnT levels were increased.

Patient 7 (SSc) was a 52-year-old man diagnosed with dcSSc in 2023. In addition to SSc, he was diagnosed with lung emphysema associated with extensive smoking (80 pack-years), which he stopped in May 2024. He presented to our department in January 2024 with suspicion of SSc-pHI and progressive skin fibrosis despite previous therapies with hydroxychloroquine, MTX and MMF. His mRSS was 22, his CRP level was elevated and he had multiple TFRs. HRCT demonstrated progressing bibasilar reticulations in addition to his emphysema. FVC was 80%. CMR imaging demonstrated SSc-pHI with increased T1 times and ECV. His hsTnT levels were elevated, and he lost 15 kg of body weight within the previous 12 months.

Patient 8 (SSc) was a 56-year-old man diagnosed with dcSSc in 2020. He developed progressive skin fibrosis, ILD and SSc-pHI. He was admitted to our department in August 2024 after failure of MMF, MTX, nintedanib and RTX (last infusion 5 months before baseline, still B cell depleted in peripheral blood at baseline). His mRSS was 28, and he had TFRs and progressive pulmonary fibrosis on HRCT with an FVC of 52%. CMR imaging showed increased T1 values, ECV and LGE; hsTnT levels were elevated. He gradually lost 10 kg of body weight over the previous 4 years.

Patient 9 (SSc) was a 46-year-old man diagnosed with dcSSc in 2022. Skin fibrosis progressed despite treatment with MTX, cyclophosphamide and RTX (last infusion 7 months before baseline; B cells partially depleted in peripheral blood). He was admitted to our department in 2024 with an mRSS of 38 and multiple TFRs. An HRCT scan demonstrated new onset of SSc-ILD (after a negative HRCT 6 months prior) with a rapid decline in FVC to 68%. His CMR imaging demonstrated new onset of biventricular systolic dysfunction (left ventricular ejection fraction (LV-EF) of 41% and right ventricular ejection fraction (RV-EF) of 37%), globally increased native T1 and T2 values as well as diffuse patchy LGE (after almost normal CMR imaging 6 months prior). Furthermore, he presented with a new left ventricular hemiblock and supraventricular tachycardia and increased hsTnT levels. Moreover, he gradually lost 9 kg of body weight within 2 years.

Patient 10 (SSc) was a 46-year-old man diagnosed with dcSSc in 2024. He also fulfilled the criteria for rheumatoid arthritis. He developed progressive skin fibrosis and progressive SSc-ILD and SSc-pHI despite previous treatment with MMF, cyclophosphamide, nintedanib and RTX (last infusion 4 months before baseline, still B cell depleted in peripheral blood). He was admitted to our department in 2025 with an mRSS of 23, multiple TFRs, rapid declines in FVC (54%) and DLCO and evidence of SSc-pHI with globally increased native T1 values on CMR imaging and elevated hsTnT levels. Moreover, he had active myositis of the pelvic and quadriceps muscles as demonstrated by MRI and muscle biopsy with elevated CK levels. He lost 18 kg of body weight since his diagnosis of SSc.

### Induction therapy with blinatumomab and teclistamab

Blinatumomab or teclistamab therapy was offered via a compassionate use program for critically ill patients according to the Arzneimittelgesetz Section 21/2 and the Arzneimittel-Härtefall-Verordnung Section 2, which allows experimental treatment if (1) patients are afflicted by a severe, potentially life-threatening disease, (2) patients have failed on previous standard-of-care treatments and (3) a scientific rationale exists—that the respective treatment potential might be efficacious in the disease. Interventions were reported to the legal authorities. Use of patient data and biomaterial from this study was covered by licenses 2022-2189 and 2023-2561 of the institutional review board of the Heinrich-Heine University of Düsseldorf. All procedures were performed in accordance with the Good Clinical Practice guidelines of the International Council for Harmonization. All participants gave written informed consent for all the procedures and the data sharing according to CARE guidelines and in compliance with the principles of the Declaration of Helsinki. No commercial sponsor was involved.

In all patients, classical and targeted synthetic and biologic DMARDs and antifibrotics were discontinued at least 1 week prior to the first dose of blinatumomab and teclistamab. Blinatumomab and teclistamab were applied as part of compassionate use programs. Patients received no compensation.

Blinatumomab has a short half-life of approximately 2.1 hours^38^ and was administered by continuous intravenous infusion in a dosage of 9 µg per day for 5 days, followed by 14 µg per day for 7 days (in total, 143 µg per cycle) (Extended Data Fig. 1). Data show the rapid decline of B cells after infusion: 50% after the first hour and 90% after 4 hours, leading to undetectable B cells after 2 days^39^. Treatment was started in an inpatient setting. After day 7, the therapy was continued in an outpatient setting. Teclistamab was subcutaneously injected at doses of 0.06 mg kg^−1^, 0.3 mg kg^−1^ and 1.5 mg kg^−1^ every other day in an inpatient setting with potential extension of the intervals up to 9 days, followed by two further cycles of teclistamab at 1.5 mg kg^−1^, each administered at 2-week intervals (Extended Data Fig. 1). Premedication for both TCEs followed the established protocols of the manufacturer and consisted of 1 g of paracetamol orally, 2 mg of clemastine intravenously and 20 mg or 16 mg of dexamethasone intravenously for blinatumomab and teclistamab, respectively.

### Maintenance therapy with RTX

To prevent redifferentiation of autoantibody-producing cells from CD20^+^ B cell precursors after the last application of TCE, we administered RTX in doses of 1 g per infusion. Determination of the dosing schedule for RTX was part of our compassionate use program, considering the pharmacokinetics of the TCE, the serological response and the clinical course, with adaptations based on insights gained from ongoing patients.

Patient 1 initially did not receive RTX, as her CD20^+^ B cells were still depleted at baseline due to a previous application of RTX. In patients 2, 3, 4 and 5, we administered RTX after the first cycle of blinatumomab, in part based on the experiences in patient 1 (Fig. 1). RTX administration was repeated every 3−5 months in order to maintain B cell depletion.

In patients with SSc, we administered maintenance therapy with repetitive doses of RTX starting 2 weeks after the last teclistamab dose. As this dosing schedule resulted in complete suppression of peripheral B cell counts and induced progressive decline of autoantibodies in patient 6, we applied this dosing scheme to all other patients with SSc as well (Extended Data Fig. 1)

In patients treated with teclistamab, aciclovir and cotrimoxazole were administered as infection prophylaxes, and 20−30 g of IVIG was infused when serum IgG dropped below 400 mg dl^−1^ for the first time and then every 4−6 weeks.

### Clinical assessment

All patients were monitored using standard clinical examination, laboratory parameters, pulmonary function testing (FVC and DLCO), 6MWT, NYHA classification, CMR imaging and HRCT. Gender was assessed using self-reports. Assessments of patients with ASyS included, additionally, MMT8, total score including both sides and axis, TIS, muscle MRI and muscle biopsy. Assessments of patients with SSc included, additionally, mRSS, TFR, EUSTAR-AI and skin biopsies. Anti-Jo1 and anti-topoisomerase 1 IgG antibody levels were measured by immunoblot (IgG EUROLINE; Euroimmun) and ELISA (EA 1599-960G and EA 1661-960GG; Euroimmun), respectively.

### Muscle and skin histopathology and immunofluorescence staining

Immunohistochemistry stainings were performed on sequential muscle cryosections with anti-CD3 (clone ZM45, 1:100; Zeta Corporation), anti-CD8 (clone C8/144B, 1:50; Agilent Technologies), anti-CD163 (clone ZM29, 1:200; Zeta Corporation), anti-MHC-I (clone W6/32, 1:800; Agilent Technologies) and anti-membrane attack complex C5b-9 (clone aE11, 1:50; Agilent Technologies) antibodies, using the avidin-biotin complex technique.

Immunofluorescence stainings of muscle cryosections were performed with anti-CD19 (302202, 1:50; BioLegend) antibodies. Nuclei were stained with DAPI (sc-3598, 1:800; Santa Cruz Biotechnology).

Stainings of skin paraffin sections were performed with the anti-BCMA (ab5972, 1:400; Abcam) antibody. For immunofluorescence, nuclei were stained with DAPI (sc-3598, 1:800; Santa Cruz Biotechnology). For immunohistochemistry, sections were co-stained with anti-CD3 (clone ZM45, 1:100; Zeta Corporation) antibodies.

The immunohistochemistry interpretation was corroborated by at least two independent observers.

CODEX staining of skin paraffin sections was performed as previously described^39^. The primary antibodies for CODEX are listed in Supplementary Table 1. Five micrometers of skin formalin-fixed paraffin-embedded (FFPE) sections were incubated on 65 °C for 1 hour, deparaffinized by xylene and rehydrated by a series concentration of ethanol. Epitope retrieval was performed with Tris-EDTA (pH 9) using a pressure cooker. To quench the autofluorescence, the tissue sections were bleached by using high-power LED panels in the bleaching solution as previously described^40–42^. After blocking with CODEX Blocking Buffer (Akoya Biosciences), sections were incubated overnight with primary antibody cocktail in Staining Buffer (Akoya Biosciences) at 4 °C. The stained tissue was fixed and stored following the instructions provided by Akoya Biosciences.

CODEX multicycle imaging was performed using a PhenoCycler-Fusion 2.2.0 equipped with a ×20 air objective (Akoya Biosciences). The barcoded reporter oligonucleotides labeled with ATTO550 and ATTO647 (biomers.net GmbH) were used to capture up to two protein stainings each cycle. Nuclear images were obtained by DAPI staining for every cycle. Aligned multiplexed images were generated in a QPTIFF format from the PhenoCycler-Fusion system. Figure 2f and Extended Data Figs. 5–7 were generated with Phenochart 2.2.0.

### Statistics

Data analysis was performed with GraphPad Prism version 5.03.

### Declaration of generative AI and AI-assisted technologies in the writing process

During the preparation of this work, the authors used Deepl.com in order to improve language and grammar. After using this tool/service, the authors reviewed and edited the content as needed and take full responsibility for the content of the publication.

### Reporting summary

Further information on research design is available in the Nature Portfolio Reporting Summary linked to this article.

## Online content

Any methods, additional references, Nature Portfolio reporting summaries, source data, extended data, supplementary information, acknowledgements, peer review information; details of author contributions and competing interests; and statements of data and code availability are available at 10.1038/s41591-026-04238-4.

## Supplementary information

Supplementary Information Supplementary Figs. 1−10.

Reporting Summary

## Figures and Tables

**Extended Data Table 1:**
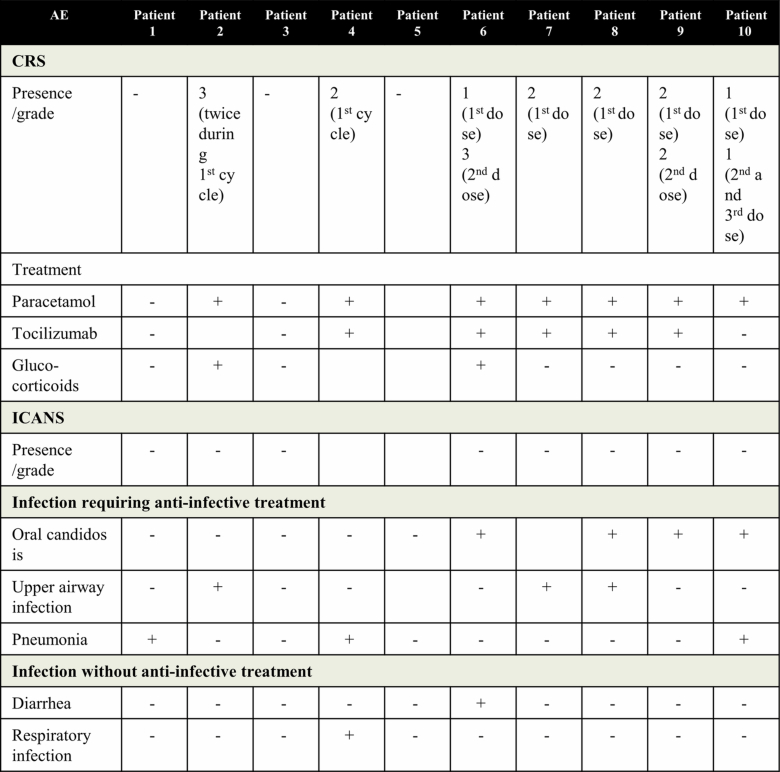
Summary of adverse events in all patients including treatment

*Abbreviations*: CRS, cytokine release syndrome; ICANS, immune effector cell-associated neurotoxicity syndrom.

## Data Availability

All data from this study are presented in the paper and figures. Individual-level clinical data are available under restricted access due to patient privacy and data protection. All requests will be reviewed for compliance with applicable institutional and regulatory requirements with responses within 2 weeks. The primary point of contact for external data requests is the corresponding author.
