# Supplementary material for: Bispecific T cell engagers for treatment-refractory autoimmune connective tissue diseases
Source: Nat Med. 2026 Feb 19;32(4):1530–42. doi: 10.1038/s41591-026-04238-4 (PMC13099420; doi:10.1038/s41591-026-04238-4)
Supplement: Supplementary file 1 — Supplementary Figs. 1−10. [file 41591_2026_4238_MOESM1_ESM.pdf]

nature medicine

Supplementary information https://doi.org/10.1038/s41591-026-04238-4

Bispecific T cell engagers for treatment-refractory autoimmune connective tissue diseases

In the format provided by the authors and unedited

Supplementary figure legends and tables

Supplementary Figure 1. MRI of affected muscles in patients with ASyS before and after induction therapy with blinatumomab and maintenance therapy with rituximab.

A Thighs of patient 1. **B** Upper arm of patient 2. **C** Thighs of patient 3. **D** Thighs of patient 4. **E** Baseline MRI of the thighs of patient 5. Follow-up not yet performed due to short follow-up time. *Abbreviations:* MRI, magnetic resonance imaging, RTX, rituximab.

Supplementary Figure 2. HRCT changes of patients with ASyS with interstitial lung disease before and after treatment with blinatumomab and rituximab.

A, B, C Comparison of HRCT findings before and after blinatumomab/RTX in patients 1, 2 and 4, respectively. **D** Baseline HRCT in patient 5; a follow-up HRCT is not yet available due to short follow-up time. *Abbreviations:* HRCT, High-Resolution computed tomography, RTX, rituximab.

Supplementary Figure 3. Comparison of the Mycobacterium kansasii-induced skin ulcer on the right Achilles' tendon of patient 3 before and 9 months after blinatumomab/RTX.

Supplementary Figure 4. Changes of the mRSS in individual patients with SSc over time after induction therapy with teclistamab and maintenance therapy with rituximab.

A – E Treatment history and changes in mRSS after teclistamab and RTX in patients 6 – 10. *Abbreviations:* mRSS, modified Rodnan skin score; RTX, Rituximab.

Supplementary figure 5. CT grading of SSc-ILD before and after treatment with teclistamab.

A CT scans before baseline and during follow-up on month 6 (patients 8 and 6) or month 3 (patient 7). Representative color-coded coronal images of apical, central and basal areas are shown (from left to right). Green indicates normal lung tissue, orange ground-glass opacities and red fibrotic/reticular lesions, as determined based on a scoring algorithm[18]. The areas that appear yellow in patient two correspond to cysts as a morphological correlate of lung emphysema. **B** Quantification of fibrosis and ground glass opacities on CT (FIBI, GGOI). *Abbreviations*: FIBI, fibrosis index, GGOI: ground glass opacity index.

Supplementary Figure 6. Muscle MRI images of patient 9 with active myositis before and after treatment with teclistamab/RTX.

Changes in myositis in patient 9 on MRI before and after teclistamab. *Abbreviations*: MRI; magnetic resonance tomography. RTX; rituximab.

Supplementary Figure 7. Changes in cardiovascular magnetic resonance tomography in patients with SSc treated with teclistamab/RTX. T1 and T2 mapping at baseline and at the end of follow-up (FU) in patient 6 (**A**), patient 7 (**B**) and patient 8 (**C**). The color-coded times in ms are given on the scale on the right. *Abbreviations*: FU; follow-up, ms; milliseconds.

Supplementary Figure 8. Changes in cardiac parameters in patients with SSc in response to treatment with teclistamab/RTX.

Changes in cardiac MRI parameters before (baseline, BL) and after teclistamab and RTX (follow-up, FU) in patients 6 – 10. *Abbreviations:* BL, baseline; FU, follow-up; ECV, extracellular volume; LV, left ventricle; LGE, late gadolinium enhancement; LVEF, left ventricular ejection fraction.

Supplementary Figure 9. Immunohistochemistry stainings of BCMA^{+} cells and CD3^{+} cells in the skin of patients 6-10 with SSc before and after teclistamab/RTX treatment. Representative immunohistochemistry stainings with slight counterstaining with hemalum are shown at magnification x200. Images marked with a black rectangle are x400 magnification of the area indicated in the lower magnification image. Scale bars = 50 µm.

Supplementary Figure 10. Co-detection by indexing (CODEX)-based imaging of B cells and plasma cells in the skin of patients with SSc before and after teclistamab. A: Representative CODEX images of CD19, CD20, CD38, CD138 and CD3 in the skin of patient 8 at baseline and 3 months after the initiation of teclistamab. **B**. Quantification of the density of CD138^{+} plasma cells in the skin of patients 7, 8, and 9 at baseline and after initiation of teclistamab.

Supplementary Table 1. Antibodies used for CODEX analysis.

Target	Clone	Source	Identifier
E-cadherin	4A2C7	Invitrogen	33-4000

Cytokeratin	C11	Biolegend	628602
CD31	C31.3 + C31.7 + C31.10	Novus Biologicals	NBP2-47785
CD45	2B11 + PD7/26	Novus Biologicals	NBP2-34528
CD3	MRQ-39	Cell Marque	103R
CD20	rIGEL/773	Novus Biologicals	NBP2-54591
CD38	E7Z8C	Cell Signaling Technology	43382SF
CD138	B-A38	Novus Biologicals	NBP3-14568

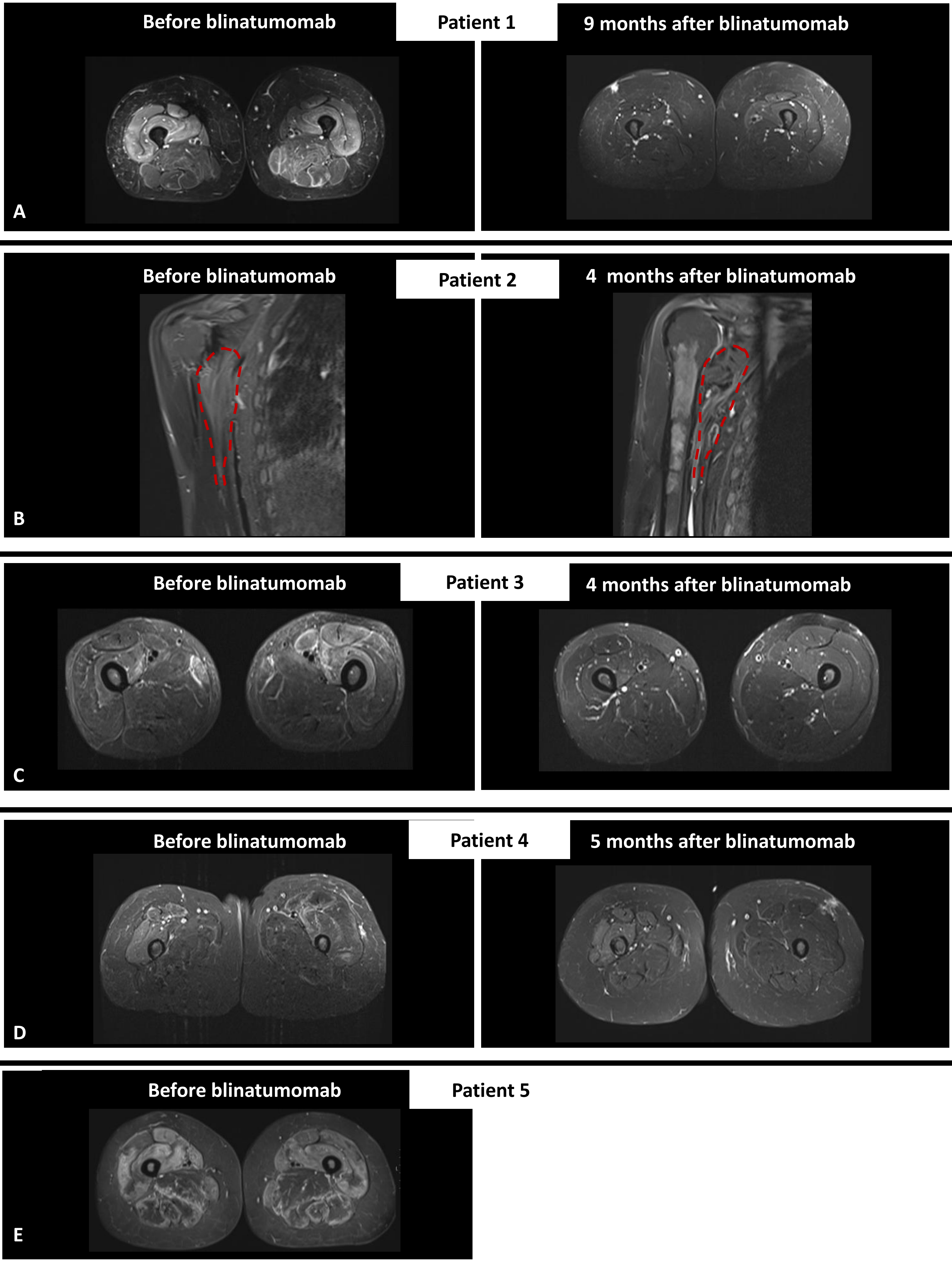
Patient 1
Before blinatumomab
9 months after blinatumomab
A
Patient 2
Before blinatumomab
4 months after blinatumomab
B
Patient 3
Before blinatumomab
4 months after blinatumomab
C
Patient 4
Before blinatumomab
5 months after blinatumomab
D
Patient 5
Before blinatumomab
E

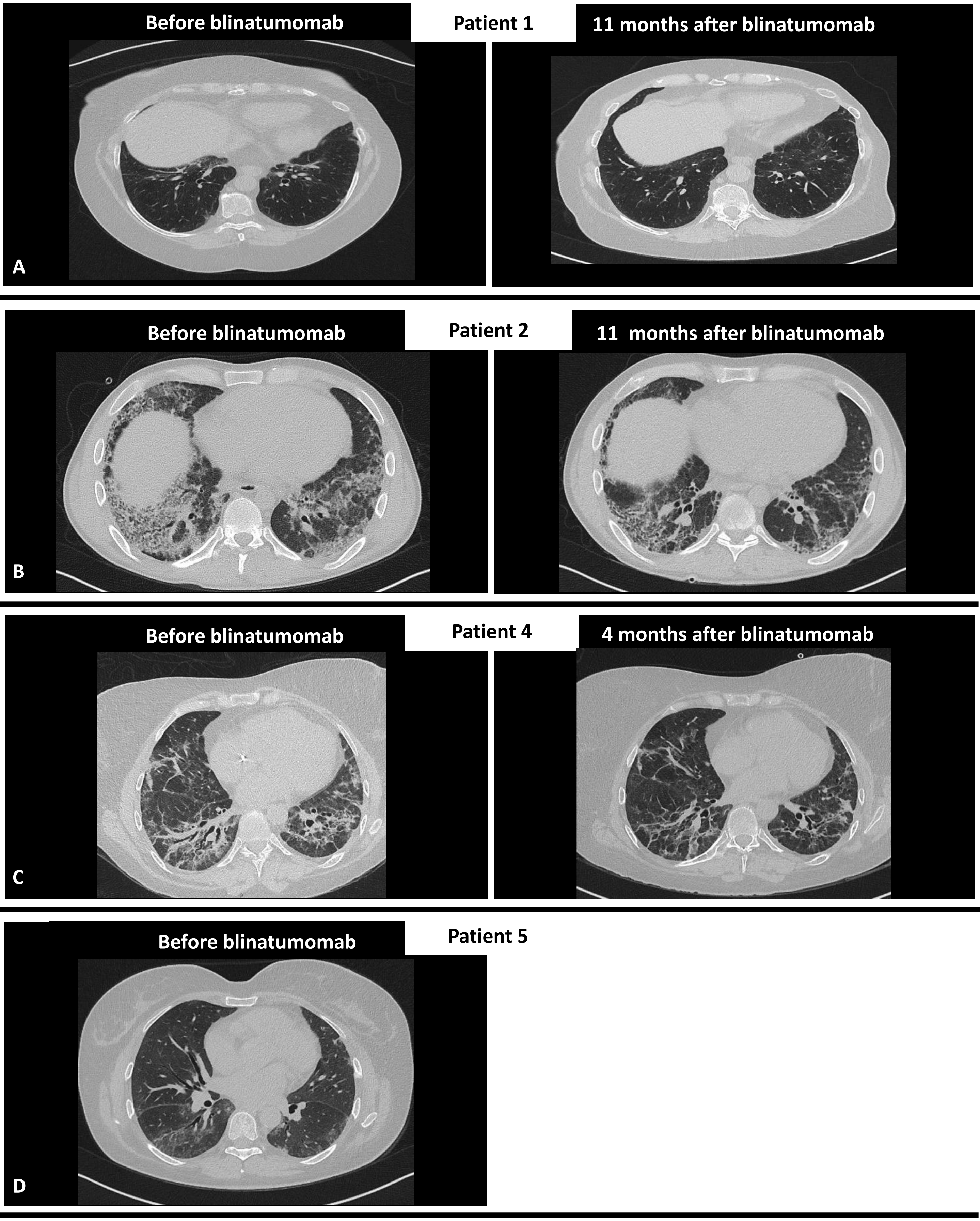
Patient 1
Before blinatumomab
11 months after blinatumomab
A
Patient 2
Before blinatumomab
11 months after blinatumomab
B
Patient 4
Before blinatumomab
4 months after blinatumomab
C
Patient 5
Before blinatumomab
D

Patient 3

Before blinatumomab | 9 months after blinatumomab

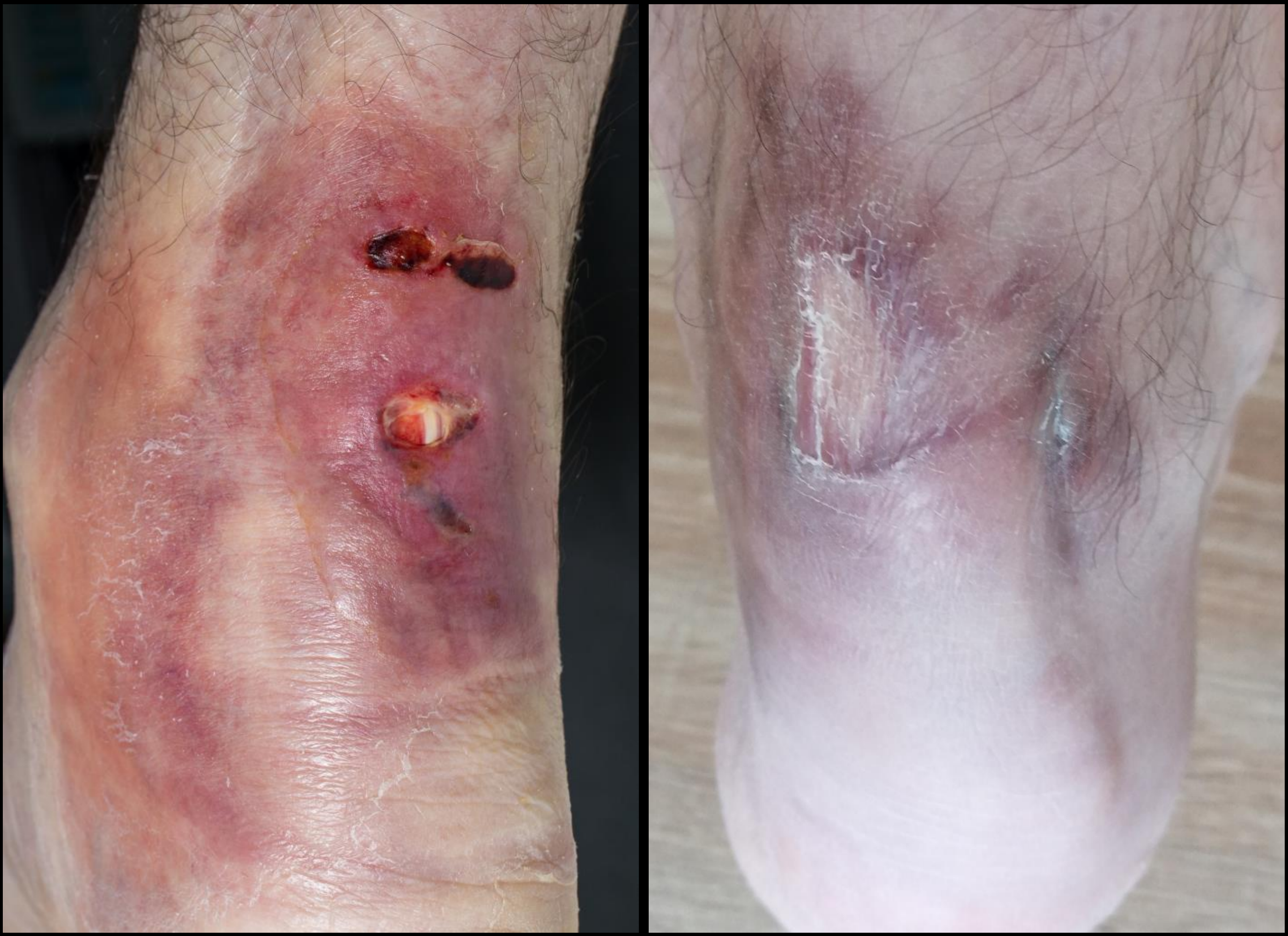

Teclistamab in SSc

A **Patient 6**

Previous treatments:
Azathioprine
Cyclophosphamide
Mycophenolate

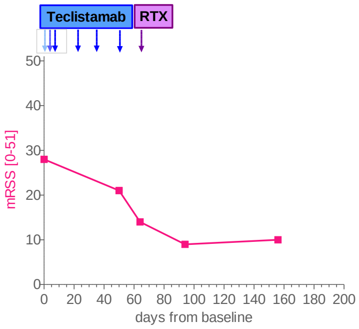

B **Patient 7**

Previous treatments:
Hydroxychloroquine
Methotrexate
Mycophenolate

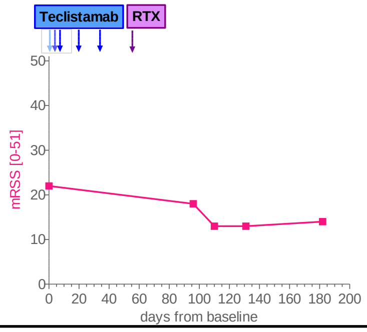

C **Patient 8**

Previous treatments:
Methotrexate
Mycophenolate
Nintedanib
RTX

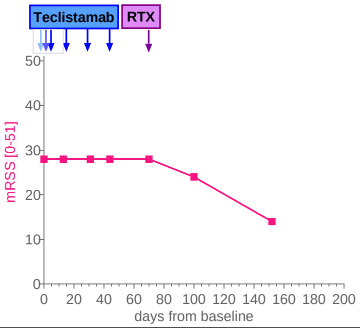

D **Patient 9**

Previous treatments:
Methotrexate
Cyclophosphamide
RTX

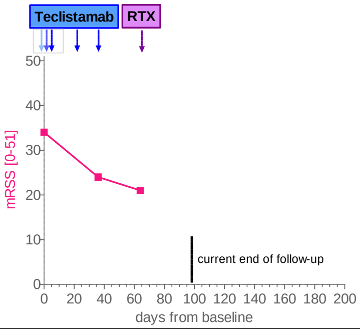

E **Patient 10**

Previous treatments:
Mycophenolate
Cyclophosphamide
Nintedanib
RTX

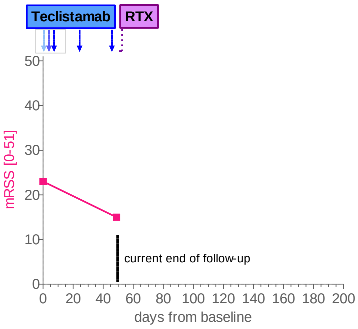

A

B

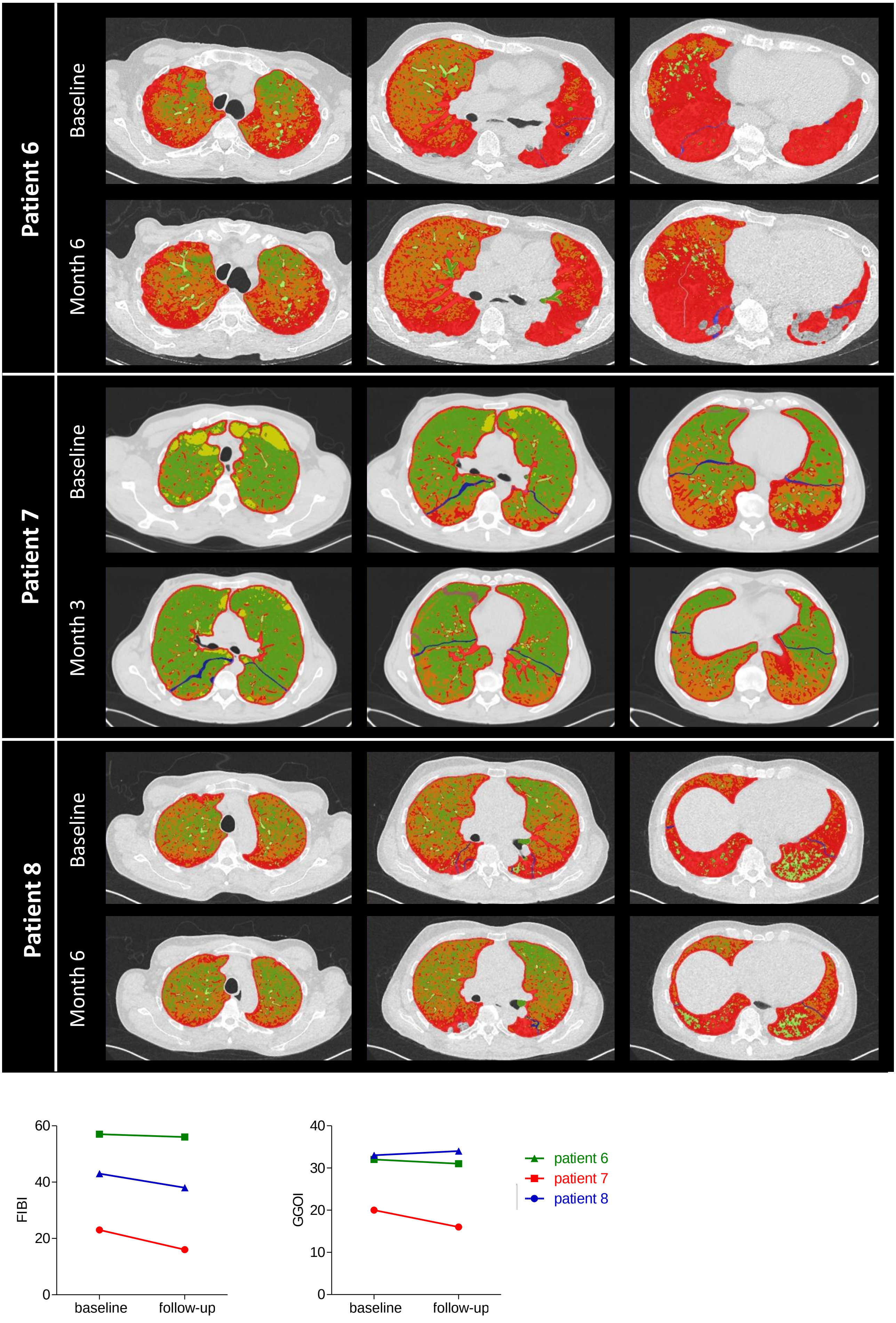

Patient 6
Baseline
Month 6
Patient 7
Baseline
Month 3
Patient 8
Baseline
Month 6
FIBI
60
40
20
0
baseline
follow-up
GGOI
40
30
20
10
0
baseline
follow-up
patient 6
patient 7
patient 8

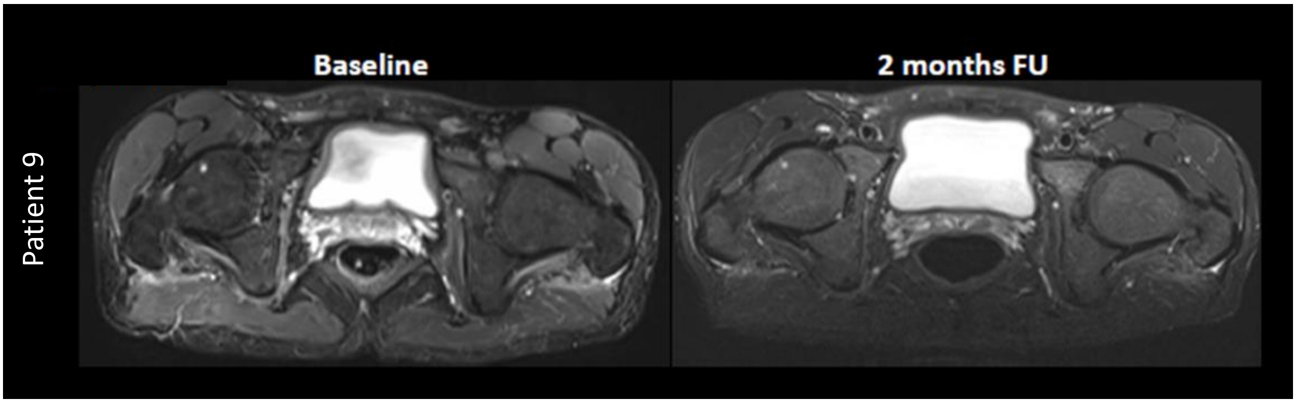
Baseline
2 months FU
Patient 9

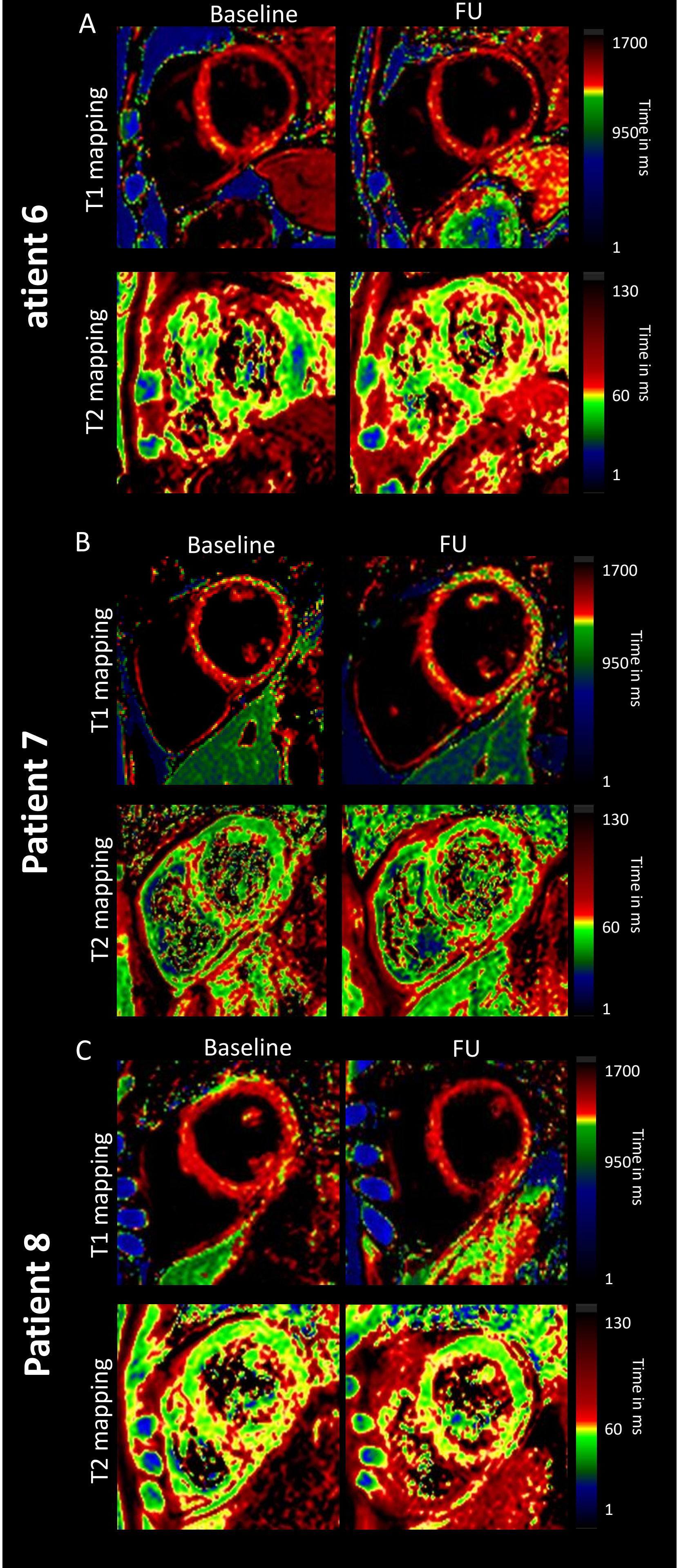

A
Baseline
FU
atient 6
T1 mapping
T2 mapping
1700
950
1
Time in ms
130
60
1
Time in ms
B
Baseline
FU
Patient 7
T1 mapping
T2 mapping
1700
950
1
Time in ms
130
60
1
Time in ms
C
Baseline
FU
Patient 8
T1 mapping
T2 mapping
1700
950
1
Time in ms
130
60
1
Time in ms

Cardiac MRI	Patient 6		Patient 7		Patient 8		Patient 9	Patient 10
	**BL**	**FU**	**BL**	**FU**	**BL**	**FU**	**BL**	**BL**
**T1 (ms)**	1384	1445	1346	1316	1351	1371	1200	1361
**T2 (ms)**	49	53	58	57	56	54	85	N/A
**ECV (%/LV)**	32	36	38	32	-	33	N/A	N/A
**LGE**	absent	absent	absent	absent	present	present	present	N/A
**LVEF (%)**	57	51	61	59	63	51	41	54

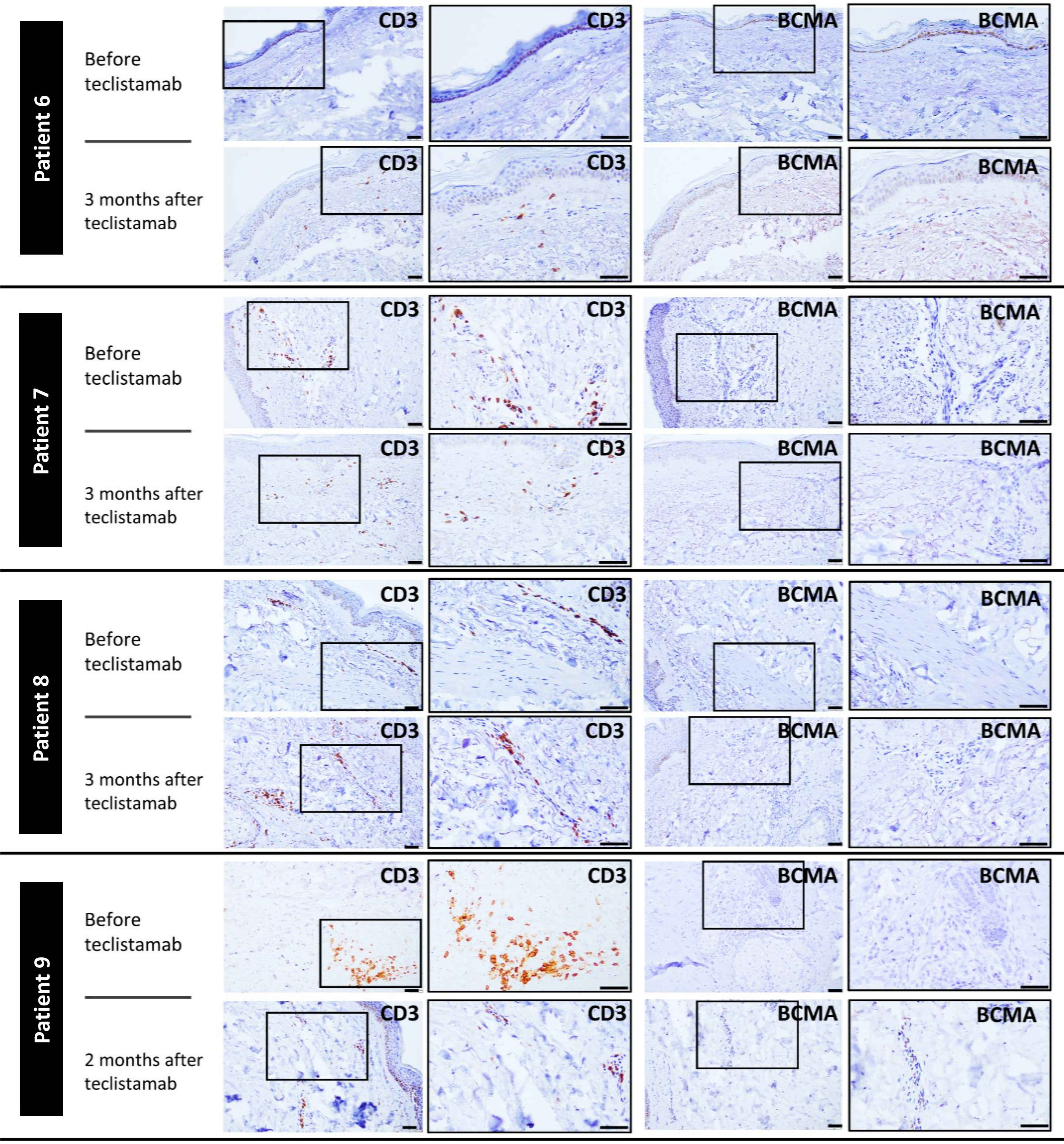
Patient 6
Before teclistamab
3 months after teclistamab
Patient 7
Before teclistamab
3 months after teclistamab
Patient 8
Before teclistamab
3 months after teclistamab
Patient 9
Before teclistamab
2 months after teclistamab
CD3
BCMA

A

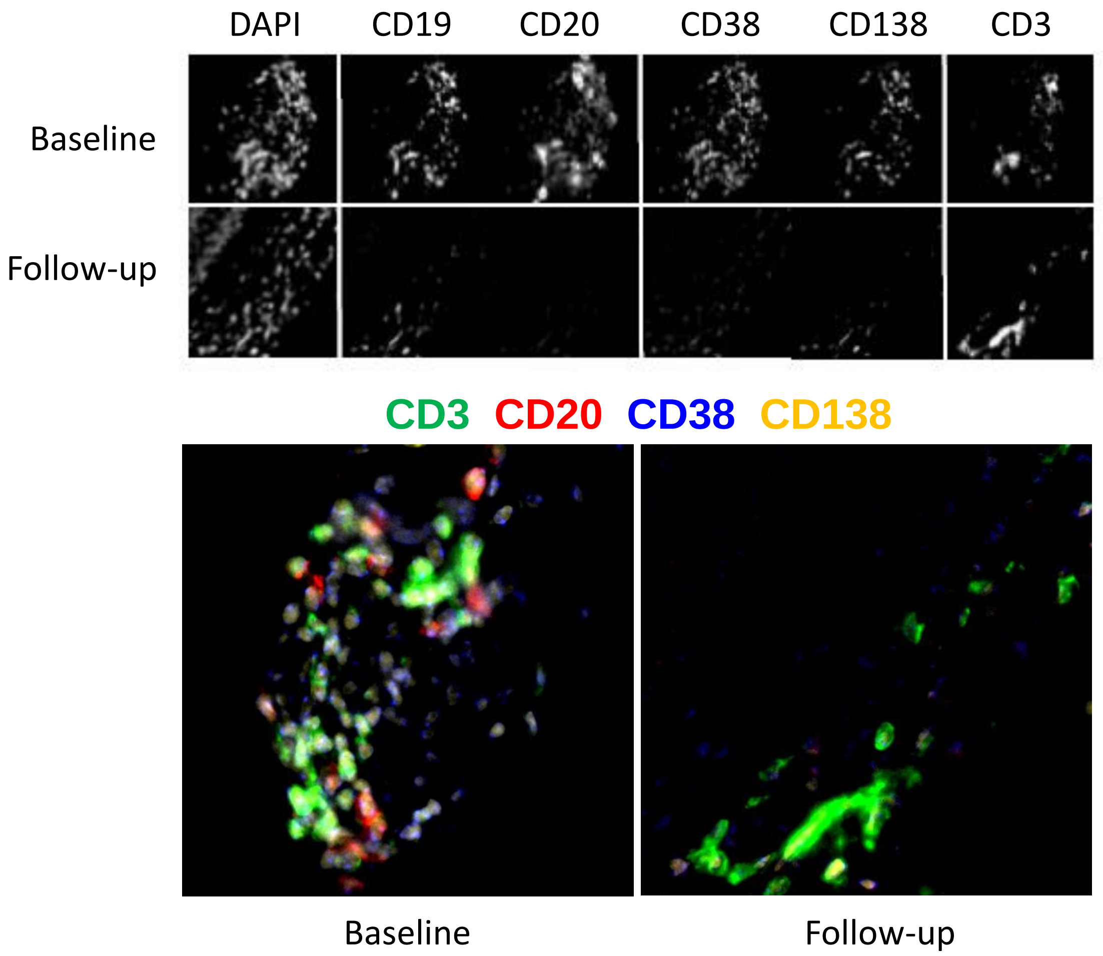
DAPI
CD19
CD20
CD38
CD138
CD3
Baseline
Follow-up
CD3 CD20 CD38 CD138
Baseline
Follow-up

B

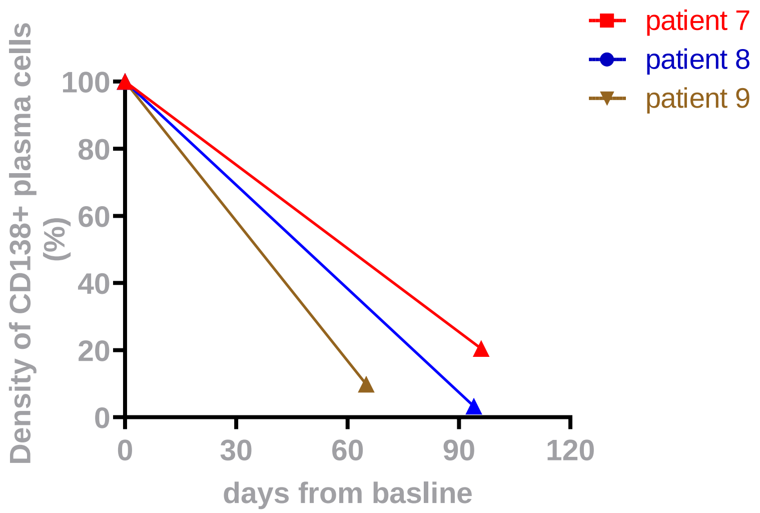
patient 7
patient 8
patient 9
Density of CD138+ plasma cells (%)
100
80
60
40
20
0
0
30
60
90
120
days from basline
